# PLK1 Inhibition Induces Synthetic Lethality in Fanconi Anemia Pathway–Deficient Acute Myeloid Leukemia

**DOI:** 10.1158/2767-9764.CRC-24-0260

**Published:** 2025-04-21

**Authors:** Aditya S. Sheth, Ka-Kui Chan, Sheng Liu, Jun Wan, Steve P. Angus, Steven D. Rhodes, Dana K. Mitchell, Christopher Davis, Maya Ridinger, Peter J. Croucher, Amer M. Zeidan, Aruna Wijeratne, Shaomin Qian, Ngoc Tung Tran, Elizabeth A. Sierra Potchanant

**Affiliations:** 1 Department of Pediatrics, Herman B Wells Center for Pediatric Research, Indiana University School of Medicine, Indianapolis, Indiana.; 2 Department of Medical and Molecular Genetics, Indiana University School of Medicine, Indianapolis, Indiana.; 3 Cardiff Oncology, San Diego, California.; 4 Yale University and Yale Cancer Center, New Haven, Connecticut.; 5 Department of Biochemistry and Molecular Biology, Indiana University School of Medicine, Indianapolis, Indiana.

## Abstract

Overall survival of acute myeloid leukemia (AML) remains limited. Inhibitors of the master mitotic kinase PLK1 have emerged as promising therapeutics, demonstrating efficacy in an undefined subset of patients with AML. However, the clinical success of PLK1 inhibitors remains hindered by a lack of predictive biomarkers. The Fanconi anemia (FA) pathway, a tumor-suppressive network comprised of at least 22 genes, is frequently mutated in sporadic AML. In this study, we demonstrate that FA pathway disruption sensitizes AML cells to PLK1 inhibition. Mechanistically, we identify novel interactions between PLK1 and both FANCA and FANCD2 at mitotic centromeres. We demonstrate that PLK1 inhibition impairs recruitment of FANCD2 to mitotic centromeres, induces damage to mitotic chromosomes, and triggers mitotic collapse in FANCA-deficient cells. Our findings indicate that PLK1 inhibition targets mitotic vulnerabilities specific to FA pathway–deficient cells and implicate FA pathway mutations as potential biomarkers for the identification of patients likely to benefit from PLK1 inhibitors.

**Significance:**

This work demonstrates that FA pathway mutations, which are frequently observed in sporadic AML, induce hypersensitivity to PLK1 inhibition, providing rationale for a novel synthetic lethal therapeutic strategy for this patient population.

## Introduction

The Fanconi anemia (FA) pathway is critical for the preservation of genomic integrity and prevention of tumorigenesis. Biallelic germline mutation of any one of the 22 genes known to comprise the pathway causes FA, a complex heritable disorder marked by congenital malformations and high incidence of cancer during childhood (1). FA is associated with a high risk of bone marrow (BM) failure which typically evolves into acute myeloid leukemia (AML). Strikingly, the risk of leukemia is increased 600-fold for individuals with FA (2). However, germline monoallelic mutations within FA genes are more frequent, a subset of which predispose to various types of malignancies (3–7). Most recognized among these carrier predispositions is the breast cancer susceptibility associated with pathogenic variants of *BRCA1* and *BRCA2* (8–10)*.* Further affecting the general population, somatic disruptions of the FA pathway are found in 30% of all sporadic cancers and more than 14% of sporadic AMLs (1, 11). Thus, FA gene function is critical for the prevention of heritable and sporadic cancers, especially AML.

The best characterized function of the FA pathway is in DNA damage detection and homologous recombination–mediated DNA damage response (DDR) during interphase (12–14). However, emerging evidence indicates that FA signaling functions throughout the cell cycle to prevent genomic instability, including in mitosis in which FA proteins are shown to have a role in centrosome function (15, 16), the resolution of ultra-fine bridges (UFB; refs. 17, 18), and cytokinesis (19, 20). Among these newly identified mitotic roles, we previously demonstrated that the FA network regulates the spindle assembly checkpoint (SAC; refs. 21, 22), a complex signaling cascade that ensures faithful chromosome segregation by delaying anaphase onset until each chromosome is properly attached to the mitotic spindle (23). These findings are consistent with the gross aneuploidy, indicative of defective chromosome segregation, which is characteristic of FA cells (24). Supporting the notion that chromosome segregation errors are a driving factor of carcinogenesis in FA, we found that aneuploidy precedes hematologic malignancy in aged FA mice (25) and that further genetic impairment of the baseline SAC defect of these mice accelerates cancer onset (26). Defining the mechanisms by which FA proteins regulate chromosome segregation may reveal new therapeutic targets that will allow the development of personalized treatment strategies in individuals with FA pathway–disrupted malignancies.

To identify druggable synthetic lethal interactors of *FANCA*, we performed a kinome-wide short hairpin RNA (shRNA) screen in *FANCA−/−* patient fibroblasts (27). This unbiased approach revealed that *FANCA-*deficient cells are hyperdependent on mitotic pathways for survival. One of the most significant hits was the major mitotic regulator polo-like kinase 1 (PLK1). This finding is consistent with work from others that have demonstrated synthetic lethality between *PLK1* and the FA pathway genes *BRCA1* and, to a lesser extent, *BRCA2* (28) in solid tumors. Similarly, synthetic lethality between *PLK1* and *FANCG* was identified in a screen but remains to be functionally validated (29). These findings suggest that loss of *PLK1* is synthetic lethal to FA-deficient cells. However, it is unclear whether this synthetic lethal interaction applies to all FA pathway proteins or is restricted to a specific subset. PLK1 drives cellular proliferation, is overexpressed in a wide range of tumors, and is associated with poor clinical outcomes (30–34), making this kinase an attractive candidate for targeted cancer therapeutics and prompting the clinical development of multiple PLK1 inhibitors (31). Whereas clinical trials evaluating the efficacy of PLK1 inhibitors in the treatment of a variety of cancers did not find inclusion of these inhibitors in therapeutic regimens to be broadly efficacious, there were subsets of patients who demonstrated clinical benefit from PLK1 inhibition (31, 35–38). The most promising results come from studies evaluating the use of PLK1 inhibitors in the treatment of myelodysplastic syndrome (MDS) and AML (37–39). Combined findings from a phase Ib and subsequent phase II trial evaluating the efficacy of onvansertib, a novel and highly specific PLK1 inhibitor, in combination with low-dose cytarabine or decitabine for patients with relapsed or refractory AML demonstrated complete remission in 20% (*n* = 9/44) of patients (40, 41). These findings suggest that clinical success of PLK1 inhibitors has been limited by a lack of predictive biomarkers capable of identifying patients likely to benefit from these agents (42).

In this study, we evaluate FA pathway deficiency as a predictor of AML sensitivity to PLK1 inhibition. Building upon the findings of our kinome-wide screen and work from others, we validate the synthetic lethal interaction between FANCA and PLK1 using *in vitro* models of AML. Furthermore, we demonstrate through whole-exome sequencing (WES) of primary AML cells that FA pathway mutations are predictive of sensitivity to PLK1 inhibition. Importantly, our retrospective analysis of a clinical trial evaluating the PLK1 inhibitor onvansertib for the treatment of AML identifies evidence supporting the notion that FANCA deficiency may predict clinical response to PLK1 inhibition. Mechanistically, we demonstrate that PLK1 inhibition exacerbates the underlying mitotic defect of FANCA-deficient cells, resulting in centromeric disintegration and mitotic failure. Together, these findings implicate FA pathway mutations as clinically useful biomarkers predictive of disease response to PLK1 inhibition and identify novel mitotic interactions between the FA pathway and PLK1.

## Materials and Methods

### Cell lines and reagents

The HL60 (ATCC, Cat# CCL-240, RRID: CVCL_0002), THP-1 (ATCC, Cat# TIB-202, RRID: CVCL_0006), OCI-AML5 (DSMZ, Cat# ACC247, RRID: CVCL_1620), and RS4;11 (ATCC, Cat# CRL-1873, RRID: CVCL_0093) cell lines were gifts from B. Gaston, P. Singh, and J. Zhang (Indiana University), respectively. hTERT-RPE1 cells were obtained from the ATCC (Cat# CRL-4000, RRID: CVCL_4388). Lymphoblast cell line GM13021 was purchased from the Coriell Institute for Medical Research (RRID: CVCL_G045). HL60, OCI-AML5, and RS4;11 cells were cultured in Iscove’s Modified Dulbecco’s Medium (12440053, Gibco). GM13021, THP-1, and Kasumi-1 (ATCC, Cat# CRL-2724, RRID: CVCL_0589) cells were cultured in RPMI (Gibco) 1640 medium. HeLa (ATCC, Cat# CCL-2, RRID: CVCL_0030), HEK293T (RRID: CVCL_0063), and PLAT-A (RRID: CVCL_B489) cells were cultured in DMEM (11965092, Gibco). hTERT-RPE1 cells were cultured in DMEM/Nutrient Mixture F-12 (11320033, Gibco). Culture media was supplemented with 10% FBS (MIDSCI), 1% penicillin–streptomycin (Gibco), and 1% sodium pyruvate (Gibco). Kasumi-1 culture media was supplemented with 20% FBS, 1% penicillin–streptomycin, 1% sodium pyruvate, and an additional 14 mmol/L D-glucose (Sigma-Aldrich; G8769). For GM13020, media was also supplemented with 500 μmol/L β-mercaptoethanol.

Stable *FANCA*-knockout (KO) HeLa cells were generated by using CRISPR/Cas9 system (Santa Cruz; sc-403205-KO-2; ref. 27). Stable FANCA-knockdown THP-1, HL60, and Kasumi-1 cell lines were established by transducing shFANCA lentivirus plasmids (Supplementary Table S1). HeLa FANCA KO cells expressing control, FANCA wild-type (WT), FANCA-Mut(Y510S), FANCA-Mut(C625S), and FANCA-Mut(L1260P) vectors were generated using lentiviral constructs generated using VectorBuilder. Stable hTERT-RPE1 cells expressing GFP–H2B and mCherry–α-tubulin were generated using MSCV–H2B–GFP (43) and MSCV–mCherry–α-tubulin reteroviral vectors (44). These vectors were gifts from G. Wahl (Salk Institute for Biological Studies) and T. Reya (Columbia University). Inducible FANCA-knockdown RPE1 (GFP–H2B/mCherry–α-tubulin) cells were generated using EZ-Tet-pLKO-Puro-shFANCA (TRCN0000118982) plasmid. EZ-Tet-pLKO-Puro plasmid (RRID: Addgene_85966) and PLAT-A cell line were a gift from Steve Angus (Indiana University). All transduced cells unless otherwise mentioned were cultured under puromycin (2 μg mL^−1^) selection for 48 hours.

Thymidine (ChemCruz) was reconstituted in PBS and filtered with a 0.2-μm syringe filter (Thermo Fisher Scientific). Ro-3306, nocodazole, volasertib, onvansertib (Selleckchem), taxol, and proTAME (Cayman Chemicals) were reconstituted in DMSO. Doxycycline (Thermo Fisher Scientific; BP26535) was reconstituted in sterile ddH_2_O.

### Immunoblotting

Cell extracts were prepared by incubating in M-PER Mammalian Protein Extraction Reagent (Thermo Fisher Scientific) or Pierce IP Lysis Buffer (Thermo Fisher Scientific) with protease (Complete Mini, EDTA-free; Roche) and phosphatase inhibitors (Pierce Phosphatase Inhibitor Mini Tablets; Thermo Fisher Scientific). Protein concentrations were measured using Pierce BCA Protein Assay Kit (Thermo Fisher Scientific), and samples were diluted with LDS sample buffer (NuPAGE; Life technologies). Proteins were separated by electrophoresis on 4% to 12% Bis-Tris gels (Invitrogen) and transferred to polyvinylidene difluoride membranes (Millipore). Membranes were blocked with Intercept Blocking Buffer (LI-COR) and probed with primary antibodies. All primary antibodies are specified in Supplementary Table S2. Blot was probed with corresponding secondary antibodies (IRDye, LI-COR), and images were captured using Odyssey CLx imager (LI-COR, RRID: SCR_014579). IMAGE STUDIO 2.1 software (RRID: SCR_015795) was used to quantify protein levels by measuring the relative fluorescence intensities of bands.

### Cell synchronization

For mass spectrometry (MS) experiments, HeLa cells were synchronized with a single thymidine block with thymidine (2 mmol/L) solution for 18 to 20 hours. The cells were released for 5 hours and blocked again with RO-3306 (9 μmol/L) for 6 hours. Cells were released into nocodazole (100 nmol/L) or proTAME (10 μmol/L) for 2 hours. We observed 90% cell synchronization throughout the cell cycle, and cells were collected through the mitotic shake-off method (45). For immunofluorescence (IF) experiments, RPE1 cells were synchronized with a single thymidine block with thymidine (2 mmol/L) solution for 18 hours. The cells were released for 5 hours after either proTAME (10 μmol/L), volasertib (20 nmol/L), or vehicle (DMSO) was added for another 6 hours.

### Immunoprecipitation of endogenous protein

Immunoprecipitation (IP) of endogenous FANCA and FANCD2 (Supplementary Table S2) was performed using Pierce Classic Magnetic IP/Co-IP Kit (Thermo Fisher Scientific) according to the manufacturer’s instruction with certain modifications. Cells were lysed using lysis/wash buffer, and lysates were precleared using protein A/G magnetic beads for 2 hours. Primary antibody was added to the precleared lysate. The mixture was incubated with agitation overnight at 4°C. Protein A/G magnetic beads were added to the mixture and incubated for 1hr at room temperature with agitation. The beads bound to protein/antibody complexes were separated using a magnetic rack. These beads were washed sequentially with lysis/wash buffer, and protein complexes were flash-frozen for downstream MS analysis. For immunoblotting experiments, protein complexes bound to the magnetic beads were eluted by incubating the beads in sample buffer with 3% β-mercaptoethanol (Sigma; M6250) followed by separation by SDS-PAGE (Invitrogen; NP0335).

### Quantitative FANCA–IP–LC-MS/MS

Beads were submitted to the IUSM Center for Proteome Analysis where on-bead digestions were performed. A measure of 30 μL 8 mol/L urea in 100 mmol/L Tris, pH 8.5, were added, and proteins were reduced with 5 mmol/L tris(2-carboxyethyl)phosphine hydrochloride (Sigma; C4706) for 30 minutes at room temperature. The resulting free cysteine thiols were alkylated with 10 mmol/L chloroacetamide (Sigma; C0267) for 30 minutes at room temperature in the dark. Samples were diluted with 50 mmol/L Tris.HCl, pH 8.5, to a final urea concentration of 2 mol/L and treated with 1 μL PNGaseF (New England Biolabs, Cat No P0705L) for 2 hours at 35°C. Samples were then digested overnight at 35°C with 0.5 μg trypsin/Lys-C (MS grade, Promega; V5072). Digestions were quenched and acidified with formic acid (0.5% TFAv/v). Peptides were desalted on Pierce C18 spin columns as per the manufacturer’s instructions (Thermo Fisher Scientific; 89870). Peptides were dried by speed vacuum, resuspended in 50 mmol/L triethylammonium bicarbonate, and then labeled for 2 hours at room temperature with 0.25 mg of Tandem Mass Tag Pro (TMTpro) reagent (TMTpro; 44520). Labeling reactions were quenched with 0.3% hydroxylamine (final v/v) at room temperature for 15 minutes. Labeled peptides were then mixed and dried by speed vacuum. After drying, samples were fractionated using Pierce High pH Reversed-Phase Peptide Fractionation Kit (Thermo Fisher Scientific; 84858) as described above.

Nano-LC-MS/MS analyses were performed on an EASY-nLC HPLC system (SCR: 014993, Thermo Fisher Scientific, RRID: SCR_014993) coupled to an Orbitrap Eclipse mass spectrometer (Thermo Fisher Scientific, RRID: SCR_022212) with a FAIMS pro interface. Peptides were separated on a 25-cm Aurora column (IonOpticks, AUR2-25075C18A) at 400 nL/minutes with a gradient of 5% to 28% with mobile phase B [mobile phases A: 0.1% FA, water; B: 0.1% FA, 80% acetonitrile (Thermo Fisher Scientific, Cat No: LS122500)] over 160 minutes, 30% to 80% B over 10 minutes, and dropping from 80% to 10% B over the final 10 minutes. The mass spectrometer was operated in positive ion mode with 3 FAIMS compensation voltages (−45, −55, −70) and 1.3 seconds cycle time per compensation voltage. Data-dependent acquisition method with advanced peak determination and Easy-IC (internal calibrant) were used. Precursor scans (m/z 400–1,600) were done with an Orbitrap resolution of 120,000, radio frequency lens% 30, maximum inject time of 105 ms, standard AGC target, MS2 intensity threshold of 2.5e4, precursor fit threshold of 70%, and 0.7 m/z window, including charges of 2 to 6 for fragmentation with 60 seconds dynamic exclusion, and single charge state per precursor dependent scan. MS2 scans were performed with a quadrupole isolation window of 0.7 m/z, 38% higher-energy collisional dissociation collision energy, 50,000 resolution, 200% normalized AGC target, and dynamic maximum IT fixed first mass of 100 m/z. The data were recorded using Thermo Fisher Scientific Eclipse Tune (v 3.3.2782.34) software (Thermo Fisher Scientific).

Resulting RAW files were analyzed in Proteome Discover 2.5 (Thermo Fisher Scientific, RRID: SCR_014477) with a Homo sapiens as well as common contaminants (Total sequences: 21,009). Sequest HT searches were conducted with full trypsin, a maximum of two missed cleavages, precursor mass tolerance of 10 ppm, and fragment mass tolerance of 0.02Da. Static modifications used for the search were carbamidomethylation on cysteine residues, TMTpro on lysines, and TMTpro on peptide N-terminus. Dynamic modifications used for the search were oxidation of methionines and protein N-terminal acetylation. The percolator FDR was set to a strict setting of 0.01 and a relaxed setting of 0.05, and the protein FDR validator in the consensus was set to a strict 1% protein FDR cutoff and relaxed 5% protein FDR cutoff; dynamic protein terminus modifications of acetylation (N-terminus), Met-loss, or Met-loss plus acetylation (N-terminus). In the consensus workflows, data were normalized to total peptide. Coisolation thresholds of 50% and average reporter ion S/N cutoffs of 10 were used for quantification. Protein abundance–based protein ratio calculation with no imputation and ANOVA (individual proteins) was performed. The resulting normalized abundance values for each sample type, abundance ratio, log_2_(abundance ratio) values, and respective *P* values (*t* test) from Proteome Discover were exported to Microsoft Excel.

### FANCA IP-MS

After washing, beads were submitted to the IUSM Center for Proteome Analysis (RRID: SCR_025538) where on-bead digestions were performed. A measure 30 μL 8 mol/L urea in 100 mmol/L Tris, pH 8.5, were added, and proteins were reduced with 5 mmol/L tris(2-carboxyethyl)phosphine hydrochloride (Sigma; C4706) for 30 minutes at room temperature. The resulting free cysteine thiols were alkylated with 10 mmol/L chloroacetamide (Sigma; C0267) for 30 minutes at room temperature in the dark. Samples were diluted with 50 mmol/L Tris.HCl, pH 8.5, to a final urea concentration of 2 mol/L and treated with 1 μL PNGaseF (New England Biolabs, Cat No P0705L) for 2 hours at 35°C. Samples were then digested overnight at 35°C with 0.5 μg trypsin/Lys-C (MS grade, Promega; V5072). Digestions were quenched and acidified with formic acid (1% v/v).

A measure of 15 μL of each sample were desalted on a 5-cm C18 trap column Acclaim PepMap 100 (3 μm particle size, 75 μm diameter; Thermo Fisher Scientific; 64946) followed by separation on a 15-cm PepMap RSLC C18 EASY-Spray column (Thermo Fisher Scientific; ES906) using an UltiMate 3000 HPLC (RRID: SCR_019840) coupled to a Q-Exactive Plus mass spectrometer (Thermo Fisher Scientific, RRID: SCR_020556) operated in positive ion mode. The gradient used for the separation of the peptides was 5% to 35% solvent B over 60 minutes, 35% to 80% solvent B over 2 minutes, 80% to 95% solvent B over 6 minutes, and 95% to 3% solvent B for 2 minutes (solvent A: 95% water, 5% acetonitrile, and 0.1% formic acid; solvent B: 100% acetonitrile and 0.1% formic acid). A data-dependent TopN20 mass acquisition method was used with the following parameters for the MS1: mass resolution: 70,000; AGC target: 3e6; maximum IT: 100 ms; and mass scan range: 300 to 2,000 m/z. MS2 parameters were fixed as follows: first mass: 100 m/z; resolution: 17,500; AGC target: 1e5; maximum IT: 50 ms; isolation window: 4.0 m/z; normalized collision energy: 30; dynamic exclusion: 30 seconds; and charge exclusion: 1, 7, 8, >8.

Raw files were analyzed in Proteome Discover 2.4 (Thermo Fisher Scientific) with a database containing UniProt reviewed *Homo sapiens* as well as common contaminants (total sequences: 21,009). Sequest HT searches were conducted with full trypsin, a maximum of two missed cleavages, precursor mass tolerance of 10 ppm, and fragment mass tolerance of 0.02 Da. Static modifications used for the search were carbamidomethylation on cysteine residues. Dynamic modifications used for the search were oxidation of methionines, phosphorylation on serine/threonine/tyrosine, and protein N-terminal acetylation. The percolator FDR was set to a strict setting of 0.01 and a relaxed setting of 0.05, and the protein FDR validator in the consensus was set to a strict 1% protein FDR cutoff and relaxed 5% protein FDR cutoff. Search results were loaded into Scaffold (version Scaffold_4.8.9, Proteome Software Inc.) with prefiltered FDR (percolator output) for viewing and statistical analysis. A Fisher exact test on total spectral with Benjamini–Hochberg test correction was performed to compare IgG-IP and FANCA-IP samples.

### Multiplexed inhibitor bead kinome assay

HL60 and THP-1 cell lines were treated with 10 nmol/L volasertib or DMSO for 24 hours and then lysed in multiplexed inhibitor bead (MIB) lysis buffer (50 mmol/L HEPES, 150 mmol/L NaCl, 0.5% Triton X-100, 1 mmol/L EDTA, and 1 mmol/L EGTA, pH 7.5) supplemented with complete protease inhibitor cocktail (Roche) and 1% phosphatase inhibitor cocktails 2 and 3 (Sigma). Extracts were sonicated 3 × 10 seconds, clarified by centrifugation, and filtered with a syringe (0.22 μm) before quantifying protein concentration by the Bradford assay. Equal amounts of total protein were gravity-flowed over MIB columns in high-salt MIB lysis buffer (1 mol/L NaCl). The MIB columns consisted of a 125 μL mixture of six type I kinase inhibitors: VI-16832, PP58, purvalanol B (46), UNC-00064-12, UNC00064-79, and BKM-120, which were custom synthesized with hydrocarbon linkers and covalently linked to ECH-Sepharose (47–50). Bound proteins were eluted twice with 0.5% SDS, 1% β-mercaptoethanol, and 100 mmol/L Tris-HCl, pH 6.8, for 15 minutes at 100°C and then treated with dithiothreitol (5 mmol/L) for 25 minutes at 60°C and 20 mmol/L iodoacetamide for 30 minutes in the dark. Following concentration by centrifuging with Amicon Ultra-4 filters (10-kDa cutoff) to ∼100 μL, samples were precipitated with methanol–chloroform, dried in a SpeedVac, and resuspended in 50 mmol/L HEPES (pH 8.0). Tryptic digests were performed overnight at 37°C, extracted four times with 1 mL ethyl acetate to remove detergent, and dried, and peptides were passed through C18 spin columns according to the manufacturer’s protocol (Pierce). Peptides were resuspended in 0.1% formic acid, and equal volume (approximately 20%–0% of the final peptide suspension for each experimental set) was injected onto a Thermo EASY-Spray 75 μm × 25 cm C18 column and separated on a 120 minutes gradient (2%–40% acetonitrile) using an EASY-nLC 1200 instrument. The Thermo Orbitrap Exploris 480 MS ESI parameters were as follows: full scan – resolution 120,000, scan range 375 to 1,500 m/z, radio frequency lens 40%, AGC target - custom (normalized AGC target, 300%), 60 seconds maximum injection time, filters – monoisotopic peak determination: peptide, intensity threshold 5.0e^3^, charge state 2 to 5, dynamic exclusion 30 seconds, data-dependent mode – 20 dependent scans, ddMS2 – isolation window 2 m/z, higher-energy collisional dissociation collision energy 30%, resolution 30,000, AGC target – custom (100%), and maximum injection time 60 seconds. Raw files were processed for label-free quantification (LFQ) by MaxQuant (version 1.6.11.0, RRID: SCR_014485) LFQ using the UniProt/Swiss-Prot human database with fixed carbidomethyl (C) and variable oxidation (M) and acetyl (protein N-terminal) modifications, with a match time window of 3 minutes. LFQ intensities for all annotated kinases with at least two razor + unique peptides were imported into Perseus software (version 1.6.10.50; RRID: SCR_015753), log_2_-transformed, and filtered to include annotated kinases (from kinome.org) with at least three valid values in one treatment group, and missing values were imputed from the normal distribution for each column using default parameters (width 0.3, down shift 1.8). Two-sample unpaired Student *t* tests of log_2_LFQ intensities were performed and plotted using R (*P* < 0.05 significance cutoff). The MS proteomics data have been deposited to the ProteomeXchange Consortium via the PRIDE partner repository (RRID: SCR_003411; ref. 51).

### IF and proximity ligation assays

Cells were grown on glass coverslips (Thermo Fisher Scientific) under indicated conditions. Cells were fixed with PFA-Triton X-100 fixation buffer (250 mmol/L HEPES, 0.2% Triton X-100, 4% methanol-free PFA, and 1× PBS, pH 7.4) at 4°C for 30 minutes or with PFA fixation buffer (4% methanol-free paraformaldehyde, 1× PBS) at room temperature for 15 minutes. Cells were washed with 1× PBS and permeabilized with blocking buffer (0.5% Triton X-100, and 5% BSA/PBS) for 30 minutes or for 1 hour. Cells were incubated in primary antibodies (1:100; Supplementary Table S2) in 5% BSA/PBS at 4°C overnight. Cells were then washed with wash buffer (0.05% Tween, 1× PBS) three times and probed with fluorophore-conjugated secondary antibodies (1:2,000 in 1× PBS) for 1hr at room temperature. The coverslips were mounted with ProLong Diamond Antifade Mountant with 4',6-diamidino-2-phenylindole (Invitrogen) on slides (Thermo Fisher Scientific). Proximity ligation assays (PLA) were performed according to the manufacturer’s instructions (Sigma; Duolink; DUO92101-1KT).

The fluorescence signals were captured by a Deltavision Ultra microscope using 60× or 100× lenses (GE Healthcare). Images were acquired with z-section of 0.2 μm each and deconvolved using SoftWoRx (GE Healthcare, RRID: SCR_019157). All images were processed with Imaris (Bitplane, RRID: SCR_007370).

### cBioPortal drug sensitivity analysis

Drug sensitivity (52) and mutation data were obtained from the Cancer Cell Line Encyclopedia (53) available through cBioPortal (RRID: SCR_014555; refs. 11, 54). IC_50_ (μmol/L) values of Fanconi pathway WT and mutant cancer cell lines (Supplementary Dataset S3) for PLK1 inhibitors BI-2536 and GW843682X (52) were compared via an unpaired Student *t* test after removing outliers.

### CD34^+^ primary AML culture

Cryopreserved mononuclear cells (MNC) derived from the BM of adult patients with AML were obtained from the Indiana University Simon Comprehensive Cancer Center Biospecimen Collection and Banking Core (RRID: SCR_025529). CD34^+^ cells were isolated using EasySep Human Cord Blood CD34^+^ Selection Kit II (STEMCELL Technologies; 17896) according to the user manual. Isolated CD34^+^ cells were expanded using StemSpan Leukemic Cell Culture Kit (STEMCELL Technologies; 09720) for 7 days before drug treatment.

### WES and analysis

WES library preparation and sequencing: Genomic DNAs were first evaluated for their quantity and quality using Agilent TapeStation 4200. A measure of 50 ng of genomic DNA were used for library preparation. Briefly, the library preparation started with shearing input DNA into smaller fragments, followed with fragment end-repair, dA tailing, ligation of index adapters, and amplification. The libraries were then hybridized, captured, and amplified with the QIAseq Human Exome probe set following the QIAseq Human Exome Handbook (Qiagen). Each resulting captured library was quantified, and its quality was accessed by Qubit and Agilent bioanalyzers (RRID: SCR_018043). Multiple libraries were pooled in equal molarity and sequenced on an Illumina NovaSeq 6000 sequencer with 150 bp paired-end reads.

FASTQ files were aligned to the human reference genome (hg38) using Burrows–Wheeler Aligner (v. 0.7.17, RRID: SCR_010910; http://bio-bwa.sourceforge.net/) PCR duplicates were removed, and coverage metrics were calculated using Picard-tools (RRID: SCR_006525) through The Genome Analysis Toolkit (GATK, v. 4.2.6.1, RRID: SCR_001876, http://www.broadinstitute.org/gatk/; ref. 55). The GATK was used for SNP and insertion/deletion (indel) discovery according to GATK best practices (56). ANNOVAR (RRID: SCR_012821, https://annovar.openbioinformatics.org/) was used to annotate resulting variants (57). Gene Ontology and Kyoto Encyclopedia of Genes and Genomes (RRID: SCR_012773) pathway functional analyses were performed on sensitivity related genes using DAVID (RRID: SCR_001881; refs. 58, 59).

### Cell viability, colony formation, and apoptosis studies

Cell viability experiments were done using CellTiter-Glo Assay Kit (Promega), and 2,000 to 2,500 cells per well were plated in a 96-well plate. After the indicated treatment, the CellTiter-Glo reagent was added according to the manufacturer’s instructions, and the luminescent signal was measured using a Synergy H4 microplate reader (BioTek, RRID: SCR_019750). Cell apoptosis experiments were done using Caspase-Glo 3/7 Assay Kit (Promega), and 2,000 to 2,500 cells per well were plated in a 96-well plate. After the indicated treatment, the Caspase-Glo reagent was added according to the manufacturer’s instructions, and the luminescent signal was measured using a Synergy H4 microplate reader (BioTek).

For BM colony formation assays, BM cells were flushed from tibias and femurs of aged and sex-matched WT and *Fanca*^−/−^ mice on C57BL/6 strain (RRID: IMSR_JAX:000664) background as described previously (60, 61). *Fanca* ± mice were a gift from D. Wade Clapp and have been previously described (62). Clonogenic methylcellulose assays were performed in triplicate at a density of 2 × 10^4^ low-density MNCs per 35-mm plate. Cultures were established in 1% Iscove’s Modified Dulbecco Medium methylcellulose (STEMCELL Technologies; M3134), 30% FCS, glutamine (20 mmol/L), penicillin and streptomycin (200 U mL^−1^), 80 μmol/L β-mercaptoethanol (Sigma; M6250), 10% BSA, mSCF (100 ng mL^−1^), mGM-CSF (10 ng mL^−1^), hEPO (4 U mL^−1^), hIL-3 (5 ng mL^−1^), mM-CSF (10 ng mL^−1^), insulin (10 μg mL^−1^), and hemin (4 mmol/L). After 6 days of incubation at 5% CO_2_ and 5% O_2_ in a humidified chamber, colonies were scored between indicated treatment conditions. All animal studies were approved by Indiana University Institutional Animal Care and Use Committee.

### Fluorescence live-cell imaging

RPE1-Scr.Ctrl. and RPE1-shFANCA were induced with doxycycline (1 μg mL^−1^) for 48 hours. Fifteen thousand cells per quadrant were plated in a 35-mm Hi-Q4 culture dish (Ibidi). Cells were presynchronized with a single thymidine (2 mmol/L) block for 18 hours. Cells were released for 5 hours, and volasertib (15 nmol/L) was added before starting the time-lapse fluorescence imaging. The time-lapse fluorescence signals were captured using Keyence BZ-X810 microscope system (RRID: SCR_025160) with GFP/TRITC epi-filter cubes and Nikon Plan Apo 20×/0.75 objective. Images were acquired with five z-sections at 2 μm every 4 minutes for a total of 16 hours. Raw data were compiled and analyzed using Imaris (Bitplane).

### Statistical analysis and figure preparation

GraphPad Prism 8 (RRID: SCR_000306) was used to perform the statistical analyses, and a *P* value <0.05 was considered significant. *P* values < 0.05, <0.01, <0.001, and <0.0001 are denoted by *, **, ***, and ****, respectively. The type of statistical analysis performed is mentioned in figure legends of each graph. Figures were prepared using GraphPad Prism 8, Adobe Illustrator, and BioRender (RRID: SCR_018361).

### Data availability

The data generated in this study are available upon request from the corresponding author. MS proteomics data are available through the ProteomeXchange Consortium via the PRIDE partner repository (accession numbers: PXD058705, PXD048057, and PXD048014). WES data are available via the dbGaP repository (accession number: phs003966.v1).

## Results

### FANCA and PLK1 exhibit a mitosis-specific interaction at centromeres

**Figure 1:**
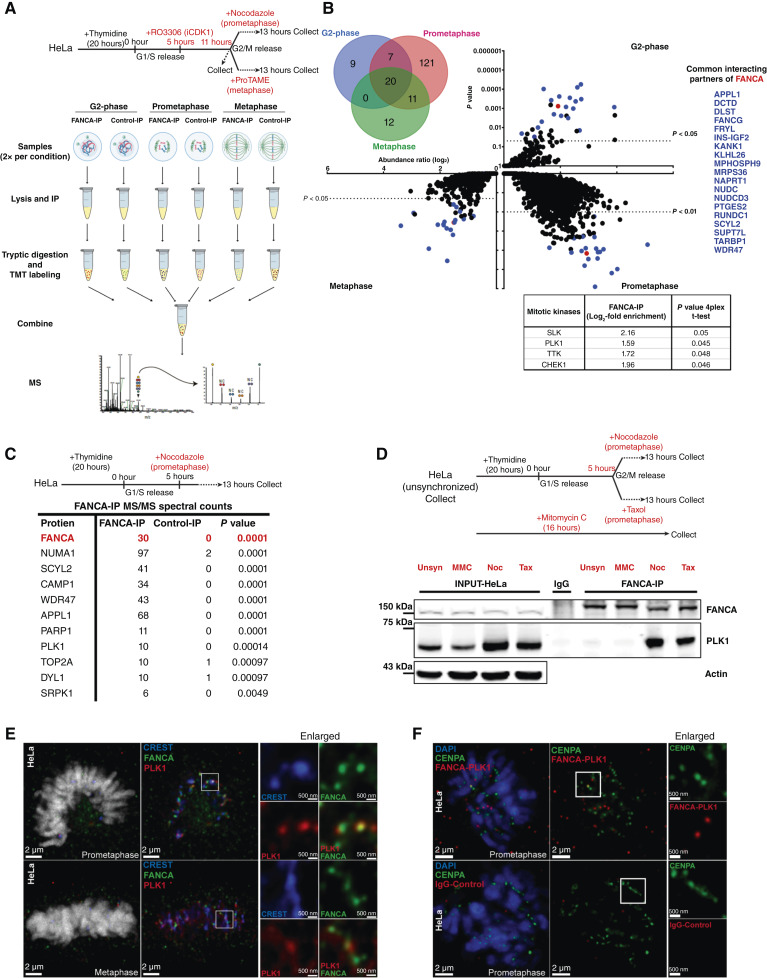
Unbiased profiling of the FANCA interactome across the cell cycle identifies PLK1 as a mitosis-specific interacting partner of FANCA. **A,** Schematic illustrating experimental design. HeLa cells were synchronized in the S-phase by a thymidine block, followed by treatment with Ro-3306 to induce G2 arrest. Cells were released from G2 into nocodazole or proTAME to arrest in prometaphase or metaphase, respectively. FANCA was immunoprecipitated from G2, prometaphase, and metaphase protein lysates (two biological replicates per condition). Precipitated FANCA immune complexes (FANCA-IP) and IgG-IP complexes were subjected to quantitative LC-MS/MS. **B,** Volcano plot of FANCA-interacting proteins identified by LC-MS/MS. A Venn diagram (top left) summarizes stage-specificity of FANCA interactions. FANCA is represented as a red dot in the volcano plots. Interacting partners enriched in all three stages of the cell cycle examined are shown as blue dots in the volcano plots and are listed in the rightmost panel (blue). Mitotic kinases enriched in prometaphase (nocodazole-treated) are listed in the bottom. Protein abundance–based protein ratios (log_2_) are represented on the *Y*-axis, and *P* values are represented on the *X*-axis. Protein abundance–based protein ratios (log_2_) and *P* values were calculated by ANOVA in Proteome Discover. **C,** Experimental design and table representing protein spectral counts from endogenous FANCA-IP vs. control IgG-IP samples from prometaphase (nocodazole-treated) synchronized HeLa cells. *P* values were calculated using the Fisher exact test with the Benjamini–Hochberg test correction in Scaffold (Proteome Software Inc.). **D,** Experimental design and a representative Western blot showing validation of FANCA–PLK1 interaction in prometaphase-enriched (nocodazole- and taxol-treated) cells. FANCA-IP was conducted in unsynchronized (Unsyn), DDR-induced (MMC-treated), and mitotic (nocodazole- and taxol-treated) HeLa cells. **E,** Representative IF images demonstrating colocalization of FANCA (green) and PLK1 (red) at centromeres (CREST, blue) in mitotic HeLa cells. Scale bars on the right and left image panels are 2 μm and 500 nm, respectively. **F,** Representative PLA images demonstrate direct interaction of FANCA and PLK1 (red) at centromeres (GFP-CENPA, green). CENPA, centromere protein A; DAPI, 4',6-diamidino-2-phenylindole; Noc, nocodazole; Tax, taxol.

To dissect the role of FANCA in mitosis, we aimed to establish a comprehensive protein interactome for FANCA in specific stages of the cell cycle. HeLa cells were synchronized in G2-phase, prometaphase, or metaphase by treatment with the CDK1 inhibitor Ro-3306, the microtubule disruptor nocodazole, or the anaphase promoting complex/cyclosome inhibitor proTAME, respectively. We then immunoprecipitated endogenous FANCA from lysates prepared from these synchronized cell populations. Next, FANCA immune complexes (FANCA-IP) were analyzed through quantitative LC-MS/MS to identify interacting proteins (Fig. 1A). This work represents the first time FANCA-interacting proteins have been interrogated in a cell-cycle phase–specific manner. Among the co-immunoprecipitated proteins identified, a total of 36 proteins showed significant enrichment with ≥2-fold abundance in G_2_-phase; 159 proteins showed significant enrichment with ≥2-fold abundance in prometaphase; and 43 proteins showed significant enrichment with ≥2-fold abundance in metaphase (Fig. 1B; Supplementary Dataset S1). Nineteen proteins were found to interact with FANCA in all three cell-cycle stages assessed (Fig. 1B right quadrant). Several mitotic kinases, including SLK, TTK, CHEK1, and PLK1, were significantly enriched in prometaphase with an increased abundance of at least 2-fold (Fig. 1B lower right quadrant). These mitotic kinases have known functions in processes such as DDR, centrosome maintenance, spindle dynamics, SAC, and cytokinesis (63–65). We performed an independent mass spectrometric analysis of endogenous FANCA immune complexes from prometaphase cells, which showed enrichment of several important mitotic proteins such as the nuclear mitotic apparatus 1, DNA topoisomerase II α, and PLK1. PLK1 was observed with 10 spectral counts in FANCA-IP versus zero spectral counts in IgG-IP (Fig. 1C). In our previous work seeking to identify druggable phosphosignaling pathways essential to the survival of *FANCA-*deficient cells, we performed a kinome-wide synthetic lethal screen in *FANCA−/−* primary patient fibroblasts compared with isogenic *FANCA*-corrected cells. This screen identified multiple mitotic kinases, including MPS1, SLK, and PLK1, as synthetic lethal targets (27). Due to the availability of clinically evaluated small-molecule inhibitors of PLK1, we prioritized the characterization of the FANCA–PLK1 interaction. To validate the specificity of the FANCA–PLK1 interaction, we evaluated the co-IP of PLK1 with endogenous FANCA from HeLa cells that were untreated, treated with mitomycin-C (MMC) to induce DNA damage, or arrested in prometaphase by nocodazole or taxol. Western blot analysis of these immune complexes revealed that FANCA–PLK1 was significantly enriched in prometaphase but not after induction of DNA damage (Fig. 1D). Specifically, we observed colocalization of FANCA and PLK1 at the centromeres during prometaphase and metaphase via IF in HeLa cells (Fig. 1E). We utilized HeLa cells stably expressing GFP-tagged centromere protein A to perform PLA-IF, which revealed that FANCA and PLK1 are no more than 40 nm apart at mitotic centromeres, further supporting a physical interaction between PLK1 and FANCA (Fig. 1F). Together, these findings demonstrate a novel interaction between FANCA and PLK1 at the centromere during mitosis.

### FA pathway disruption sensitizes cells to low concentration of the PLK1 inhibitor volasertib *in vitro*

PLK1 is an essential kinase for mitotic progression (66), and its overexpression is a common feature in several human malignancies (31, 67). Importantly, the therapeutic efficacy of small-molecular inhibitors targeting PLK1 is an ongoing area of clinical evaluation for the treatment of a range of cancers (35, 36, 38, 40). Interestingly, our retrospective analysis of the DepMap database (68, 69) revealed significantly higher expression of *PLK1* in cancers with FA pathway disruptions compared with cancers with intact FA pathway, suggesting that FA pathway disruption may lead to PLK1 “addiction” (Supplementary Fig. S1A and S1B). To broadly assess whether FA pathway mutations sensitize to PLK1 inhibitors, we utilized cancer cell line drug sensitivity data publicly available from the Cancer Cell Line Encyclopedia (RRID: SCR_013836; ref. 53). We observed significantly increased sensitivity to the PLK1 inhibitors GW843682X and BI-2536 in cancer cell lines with FA pathway mutations compared with those with WT FA genes (Supplementary Fig. S1C and S1D; Supplementary Datasets S2–S5). The most striking difference was observed in *FANCA*-mutated cancer cell lines (Supplementary Fig. S1E and S1F).

**Figure 2:**
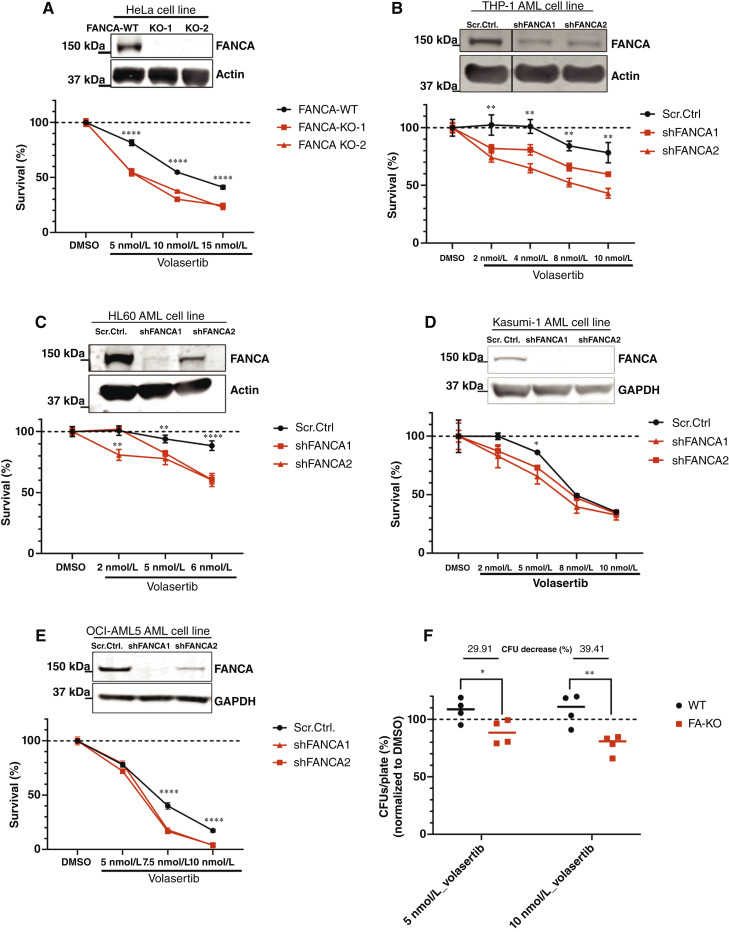
FANCA deficiency sensitizes cancer cell lines and primary murine HPCs to PLK1 inhibition. Representative Western blots showing FANCA expression in WT vs. *FANCA*-KO in HeLa (**A**) Scr.Ctrl vs. FANCA-KD THP-1 (**B**), HL60 (**C**), Kasumi-1 (**D**)**,** and (**E**) OCI-AML-5 cells. Graphs below each Western blot represent cell viability of FANCA-KD cells relative to control following 3-day treatment with volasertib. For all cell lines, viability was measured by CellTiter-Glo assay. Plots show the mean ± SEM of data from one representative experiment. All experiments were repeated independently at least 3 times. *P* values were calculated by two‐way ANOVA. **F,** Quantification of colony formation in *Fanca*-KO vs. WT BM following 6-day volasertib treatment. Representative graph shows the mean ± SEM CFUs/plate relative to vehicle. For each genotype, drug treated values are normalized to vehicle alone. *P* values were calculated by two‐way ANOVA. Each data point on the graph represents an individual mouse.

To validate that FANCA-deficient cells are hyperdependent on PLK1 for survival, we performed CRISPR-mediated KO of *FANCA* in HeLa cells. *FANCA*-WT and *FANCA*-KO HeLa cells were treated *in vitro* with low concentrations (2–15 nmol/L) of the PLK1 inhibitor volasertib. Importantly, these concentrations have been shown to spare healthy hematopoiesis while retaining high cell death–inducing efficacy in primary MDS/AML cells *in vitro* (39). We observed a significant decrease in viability of *FANCA-*KO cells compared with *FANCA*-WT cells (Fig. 2A). To further validate that loss of FANCA sensitizes AML cells to low concentration of volasertib, we stably knocked down FANCA using shRNA in four different AML cell lines: THP-1 (acute monocytic leukemia), HL60 (acute promyelocytic leukemia), Kasumi-1 (acute myeloblastic leukemia), and OCI-AML-5 (Fig. 2B–E). Additionally, we stably knocked down FANCA in the acute lymphoblastic leukemia cell line RS4;11 (Supplementary Fig. S2). Knockdown of FANCA sensitized all cell lines to low concentrations of volasertib to varying degrees, as assessed by *in vitro* cell viability assay. Kasumi-1 cells were one of the most sensitive cell line to low-dose volasertib (IC_50_ = 5–8 nmol/L), likely due to the presence of *NF1*, *NRAS*, and *KRAS* mutations, which have been shown to sensitize to PLK1 inhibition (70–72). Because of this baseline sensitivity, a significant difference between Scr.Ctrl and shFANCA was detectable only at 5 nmol/L volasertib. To assess synthetic lethality in normal hematopoietic cells, we assessed the colony-forming ability of hematopoietic progenitor cells (HPC) from the BM of WT and *Fanca*−/− mice in the presence of a low concentration of volasertib. Indeed, volasertib treatment significantly decreased the colony-forming potential of *Fanca*−/− HPCs compared with WT HPCs, in which colony formation was not impaired (Fig. 2F).

To confirm that the low concentration of volasertib used in our *in vitro* experiments selectively inhibits PLK1, we utilized MIB affinity chromatography coupled with MS (ref. 73), a comprehensive proteomic approach that quantitatively assesses the activation state of 80% to 90% of the human kinome (47, 48). This approach confirmed that PLK1 was the only kinase significantly inactivated in HL-60 and THP-1 cells treated with the low concentration of volasertib (10 nmol/L) that triggers synthetic lethality in FANCA-deficient cells (Supplementary Fig. S3A–S3D).

To confirm selective sensitivity to low concentration PLK1 inhibition, we repeated our *in vitro* cell viability assays using onvansertib, another PLK1-specific small-molecule inhibitor that has demonstrated clinical promise (40) and continues to be evaluated in ongoing clinical trials. Consistent with the results above, we found that stable knockdown of FANCA conferred hypersensitivity to onvansertib in both AML cell lines evaluated (Supplementary Fig. S4A and S4B). We also observed a modest decrease in the colony-forming potential of *Fanca*−/− murine HPCs compared with WT HPCs after onvansertib treatment (Supplementary Fig. S4C).

**Figure 3:**
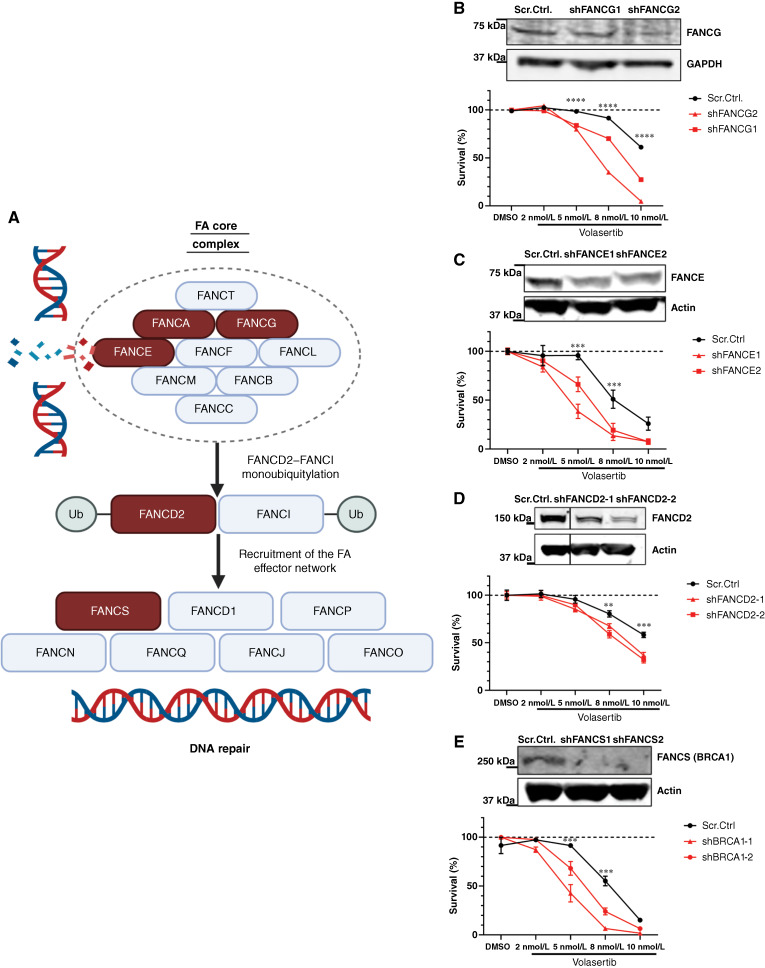
FA pathway dysfunction sensitizes AML cells line to volasertib. **A,** Illustration of the FA pathway during DDR. FANCA, FANCG, FANCE, FANCD2, and FANCS/BRCA1 are highlighted in red. Representative Western blots showing knockdown of FANCG (**B**), FANCE (**C**), FANCD2 (**D**), and FANCS (**E**) in HL60 cells. Graphs below each Western blot represent cell viability of knockdown cells relative to control following 3-day treatment with volasertib. *P* values were calculated by two‐way ANOVA. Cell viability was measured by CellTiter-Glo assay. Representative graph shows the mean ± SEM cell survival (%) compared with vehicle. All experiments were repeated a minimum of 3 times. Ub, ubiquitination.

Beyond FANCA, previous literature suggests cells deficient in FANCG and FANCS (BRCA1) to be hypersensitive to PLK1 inhibition. Thus, we broadened our evaluation of PLK1 hypersensitivity to other members of the FA pathway (Fig. 3A). We found that knockdown of FANCG, FANCE, FANCD2, and BRCA1 significantly sensitized HL60 AML cells to volasertib with varying degrees of severity (Fig. 3B–E). These findings validate FANCG- and BRCA1-PLK1 synthetic lethal interactions in the setting of AML and identify novel synthetic lethal interactions between PLK1 and two additional FA pathway members (FANCE and FANCD2).

### Damaging FA pathway mutations sensitize primary AMLs to PLK1 inhibition

**Figure 4:**
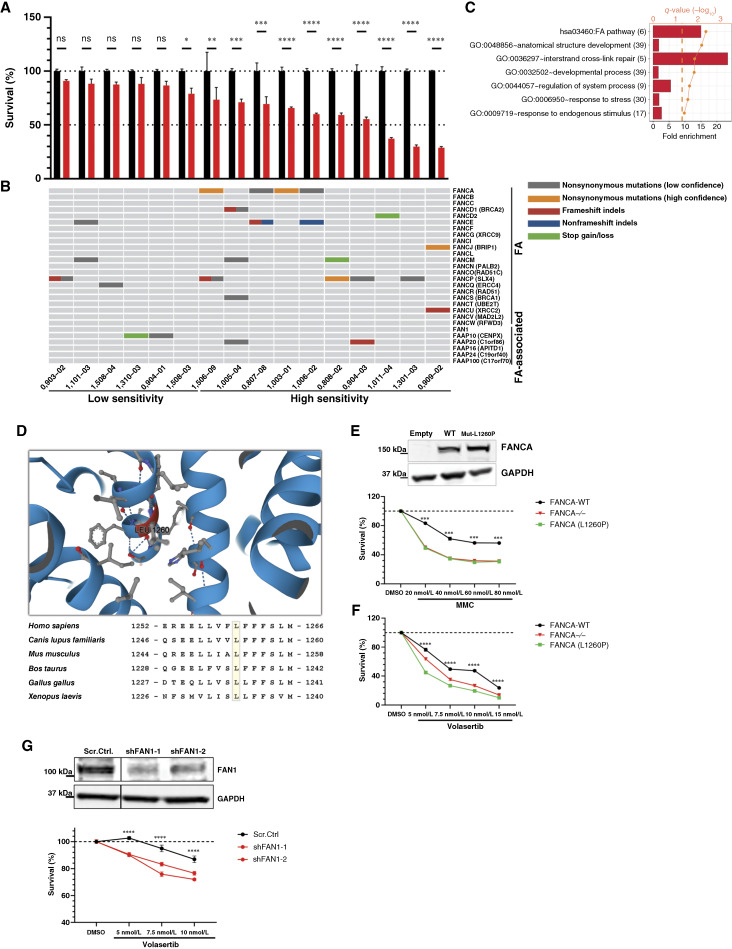
Low-dose volasertib causes selective toxicity in primary AMLs with predicted pathogenic FA pathway mutations. **A,** Graph represents viability of primary patient CD34^+^ AML cells treated with 10 nmol/L volasertib for 5 days. *P* values were calculated by two‐way ANOVA. **B,** Panel below bar graph indicates predicted pathogenic FA pathway mutations in patient AML samples identified through WES. **C,** Graph represents Gene Ontology and Kyoto Encyclopedia of Genes and Genomes pathway functional pathways that were significantly enriched among identified gene mutations (*P* value < 0.05). **D,** Top depicts the position of FANCA residue L1260 within the predicted H12A helix of the C-terminal domain. Protein structure was predicted using AlphaFold Protein Structure Database (version 2022-11-01, RRID: SCR_023662). Bottom shows conservation of L1260 residue (highlighted in yellow) across multiple species. **E,** Representative Western blot showing FANCA expression in HeLa *FANCA-*KO cell line transfected with empty vector, *FANCA*-WT, or *FANCA*-Mut(L1260P). Representative graph shows the mean ± SEM cell viability (%) of empty (*FANCA-*KO), *FANCA*-WT, and *FANCA*-Mut(L1260P) HeLa cells treated with MMC for 4 days. *P* values were calculated by two‐way ANOVA. **F,** Representative graph shows the mean ± SEM cell viability (%) of empty (*FANCA-*KO) and *FANCA*-WT vs. *FANCA*-Mut(L1260P) HeLa cells treated with volasertib for 3 days. *P* values were calculated by two‐way ANOVA. **G,** Representative Western blot of FAN1 expression in control cells (Scr.Ctrl) vs. FAN1-KD (shFAN1) HeLa cells. Representative graph shows the mean ± SEM cell viability (%) of shFAN1 cells vs. Scr.Ctrl. HeLa cells treated with volasertib for 3 days. For all cell lines, viability was measured by CellTiter-Glo assay. *P* values were calculated by two‐way ANOVA. All experiments were repeated at least 3 times. ns, not significant.

To validate our findings in primary AML, we obtained cryopreserved MNCs derived from the BM of 16 adult patients with AML from the Indiana University Simon Comprehensive Cancer Center Biospecimen Collection and Banking Core as a panel for comparative volasertib sensitivity assessment (Fig. 4A). Through WES, we identified multiple pathogenic mutations in the FA pathway and FA pathway–associated genes (Supplementary Dataset S6). These included nonsynonymous mutations, nonsynonymous mutations leading to stop loss or stop gain, frameshift indels, and nonframeshift indels. Nonsynonymous mutations identified as pathogenic by three independent prediction algorithms [SIFT (74), DEOGEN2 (75), and FATHMM (76)] were categorized as high-confidence pathogenic mutations (Fig. 4B, orange shading). Low-confidence pathogenic mutations were predicted to be pathogenic by the SIFT algorithm alone (Fig. 4B, gray shading). To compare the volasertib sensitivity of these AMLs, CD34^+^ cells were purified from all samples, expanded, and subjected to viability assays following volasertib treatment. Utilization of CD34^+^ selection enriches for leukemic stem/progenitor cell populations, which are relatively resistant to chemotherapies (77). We found that higher FA pathway mutation burden correlated with sensitivity to volasertib: low concentration of volasertib (10 nmol/L) caused a ≥25% decrease in viability for 7/8 AML samples with ≥2 FA pathway mutations compared with only 3/8 of the AML samples with ≤1 FA pathway mutations (Fig. 4A and B). Notably, Gene Ontology analysis of our sequencing data revealed that FA pathway mutations were significantly enriched in the AMLs that exhibited increased sensitivity to volasertib (Fig. 4C). We functionally tested two high-confidence *FANCA* mutations, *FANCA* c.1529A>C (p.Y510S) and *FANCA* c.1874G>C (p.C625S), observed in primary AML samples 1003-01 and 1506-09, respectively (Fig. 4B; Supplementary Dataset S6). Both residues, tyrosine 510 and cysteine 625, are highly conserved and positioned within critical helices of FANCA protein (Supplementary Fig. S5A and S5B; ref. 78). To assess the functionality of these mutant proteins, we reexpressed FANCA-WT and FANCA-Mut(Y510S) and FANCA-Mut(C625S) in *FANCA-*KO HeLa cells (Supplementary Fig. S5C). The reexpression of FANCA-WT rescued the inherent MMC hypersensitivity of the FANCA-deficient cells, whereas the FANCA-Mut(Y510S) protein did not. Interestingly, FANCA-Mut (C625S) partially rescued the MMC hypersensitivity but not to the level of FANCA-WT (Supplementary Fig. S5D). Similarly, reexpression of FANCA-WT rescued the volasertib hypersensitivity of the *FANCA*-KO cells, but the FANCA mutant proteins did not (Supplementary Fig. S5E).

To further assess the clinical relevance of these findings, we performed retrospective analysis of RNA sequencing (RNA-seq) data obtained during the phase 1b/2 clinical trials that evaluated onvansertib as combination therapy for relapsed/refractory AML (40, 41). In the phase 1b trial, onvansertib was orally administered in escalating doses to 40 patients in combination with low-dose cytarabine (20 mg.m^−2^) or decitabine (20 mg.m^−2^; ref. 40). Of patients in these two arms, 7% (*n* = 17) and 24% (*n* = 23), respectively, showed complete remission. The patient that achieved the best and most durable response, patient 07-009, received onvansertib (12 mg.m^−2^) in combination with decitabine. In this study, we report the AML mutation landscape for this patient as determined by total RNA-seq (Supplementary Table S3), which revealed heterozygous mutations in *FANCA* at c.3779T>C (p.L1260P) and the FA-associated gene *FANCD2 and FANCI-associated nuclease 1* (*FAN1*) at c.2435G>A (p.C812Y). Both mutations were predicted to be pathogenic by FATHMM. The Leucine1260 residue of FANCA is a highly conserved amino acid positioned within in the predicted H12A helix near the C-terminal domain (Fig. 4D; ref. 78). Whereas this mutation has been reported in prostate cancer and is predicted to be pathogenic (79), its impact on FANCA function has not been directly assessed. To determine whether FANCA-Mut(L1260P) mutant protein can rescue the MMC hypersensitivity of FANCA-deficient cells, we reexpressed FANCA-WT and FANCA-Mut(L1260P) in *FANCA-*KO HeLa cells [Fig. 4E (top)]. As predicted, the reexpression of FANCA-WT rescued the inherent MMC hypersensitivity of the FANCA-deficient cells, whereas the FANCA-Mut(L1260P) protein did not [Fig. 4E (bottom)]. Similarly, reexpression of FANCA-WT rescued the volasertib hypersensitivity of the *FANCA*-KO cells, but the FANCA-Mut(L1260P) protein failed to rescue this hypersensitivity (Fig. 4F). As patient 07-009 also had a predicted pathogenic mutation in *FAN1*, we asked whether loss of FAN1 also conferred volasertib hypersensitivity. Indeed, we found that FAN1-knockdown caused hypersensitivity of HeLa cells to PLK1 inhibition (Fig. 4G). Collectively, these findings corroborate synthetic lethality between PLK1 and FA pathway disruption in primary AML.

### Inhibition of PLK1 increases mitotic arrest and polyploidy in FANCA-deficient cells

**Figure 5:**
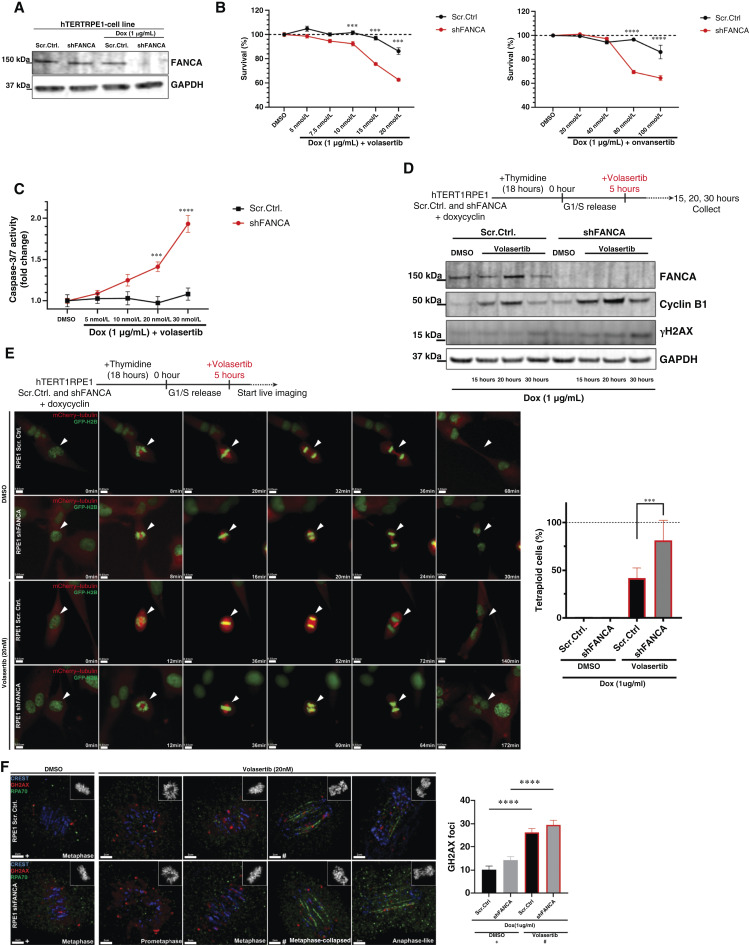
Inhibition of PLK1 induces mitotic failure and apoptosis in the FANCA-deficient hTERT-RPE1 cell line. **A,** Representative Western blot showing FANCA expression in control (Scr.Ctrl.) cells vs. FANCA-KD (shFANCA) hTERT-RPE1 cells after 48 hours of doxycycline (Dox) treatment. **B,** Concentration-dependent survival (%) of shFANCA and Scr.Ctrl cells treated with volasertib and onvansertib for 3 days. Cell viability was measured by CellTiter-Glo assay. Representative graph shows the mean ± SEM cell survival (%) compared with vehicle. *P* values were calculated by two‐way ANOVA. **C,** Concentration-dependent cellular apoptosis (fold change) of shFANCA and Scr.Ctrl. hTERT-RPE1 cells treated with volasertib for 3 days. Caspase 3/7 activity was measured by Caspase-Glo 3/7 assay. Representative graph shows the mean ± SEM caspase 3/7 activity (fold change) compared with vehicle. *P* values were calculated by two‐way ANOVA. **D,** Experimental design and a representative Western blot showing increased cyclin B1 and γH2AX in shFANCA vs. Scr.Ctrl. hTERT-RPE1 cells. **E,** Experimental design and representative live-cell imaging images of shFANCA and Scr.Ctrl. hTERT-RPE1 cells stably expressing mCherry–tubulin (red) and GFP–H2B (green) after DMSO and volasertib treatment. Scale bars in the image panels represent 0.03 μm. Representative graph showing quantification of cells undergoing mitotic collapse and cytokinesis failure (*n* = 50 cells per group). Graph shows the mean ± SEM. *P* values were calculated by one‐way ANOVA. **F,** Representative IF images show γH2AX foci (red) and UFBs (RPA70, green) in shFANCA and Scr.Ctrl. mitotic hTERT-RPE1 cells after vehicle and volasertib (20 nmol/L) treatment. Scale bars in the image panels represent 2 μm. Representative graph showing quantification of γH2AX foci (*n* ≥ 10 cells per group) in DMSO-treated metaphase (+) cells vs. volasertib-treated (20 nmol/L) metaphase-collapsed (#) cells. Graph shows the mean ± SEM. *P* values were calculated by one‐way ANOVA. All graphs are representatives of at least three independent experiments.

To define the mechanism underlying the hypersensitivity of cells with FA pathway dysfunction to PLK1 inhibition, we began by generating hTERT-RPE1 cells with CRISPR-mediated KO of *FANCA*. We chose to conduct these studies using hTERT-RPE1 cells because they are nonmalignant and can be effectively synchronized in mitosis, making them an ideal model for studying mitotic mechanisms. Therefore, we generated hTERT-RPE1 cell lines with stable integration of doxycycline-inducible shRNA targeting FANCA or scrambled control (Fig. 5A). We first tested the volasertib sensitivity of doxycycline-induced sh*FANCA* and control hTERT-RPE1 cells. We observed a concentration-dependent increase in hypersensitivity to volasertib and onvansertib in FANCA*-*knockdown cells compared with control hTERT-RPE1 cells through cell viability assay (Fig. 5B). Consistent with the decrease in cell viability, we observed an approximately 2-fold increase in caspase-3/7 activity in doxycycline-induced FANCA*-*knockdown hTERT-RPE1 cells compared with their control counterparts (Fig. 5C). Accordingly, we observed increased levels of cyclin B1 and γH2AX in doxycycline-induced FANCA*-*knockdown hTERT-RPE1 cells compared with their control counterparts following treatment with volasertib, indicating increased mitotic arrest and DNA damage, respectively (Fig. 5D). PLK1 inhibition is known to induce centromere decompaction and disintegration, causing collapse of metaphase alignment, loss of chromosome biorientation, and induction of postmitotic DNA damage (80). To test whether PLK inhibition in FANCA-depleted cells exhibited a similar phenotype, we generated doxycycline-inducible shFANCA and scrambled control hTERT-RPE1 cells stably expressing mCherry-fused α-tubulin and GFP-fused histone H2B. These cell lines were used to perform live-cell imaging in the presence of volasertib. Cells were presynchronized in the S-phase by a single thymidine block and then released into low-concentration volasertib (20 nmol/L). Live-cell imaging studies revealed that more than 80% of *FANCA*-knockdown hTERT-RPE1 cells underwent metaphase collapse and subsequent cytokinesis failure after volasertib treatment compared with 45% to 50% of control cells (Fig. 5E). Furthermore, through fixed IF imaging, we observed a significant increase in γH2AX foci and RPA70-positive UFBs in FANCA*-*knockdown cells undergoing volasertib-induced mitotic collapse relative to their control counterparts (Fig. 5F). Notably, the increase in γH2AX foci were not due to S-phase–associated DNA damage, as volasertib was introduced 5 hours after release from a single thymidine block, suggesting that the DNA damage observed was specific to mitosis. Together, these findings suggest that FANCA-deficient cells are highly susceptible to mitotic failure after PLK1 inhibition.

### FANCD2 interacts with PLK1 at centromeres during mitosis

**Figure 6:**
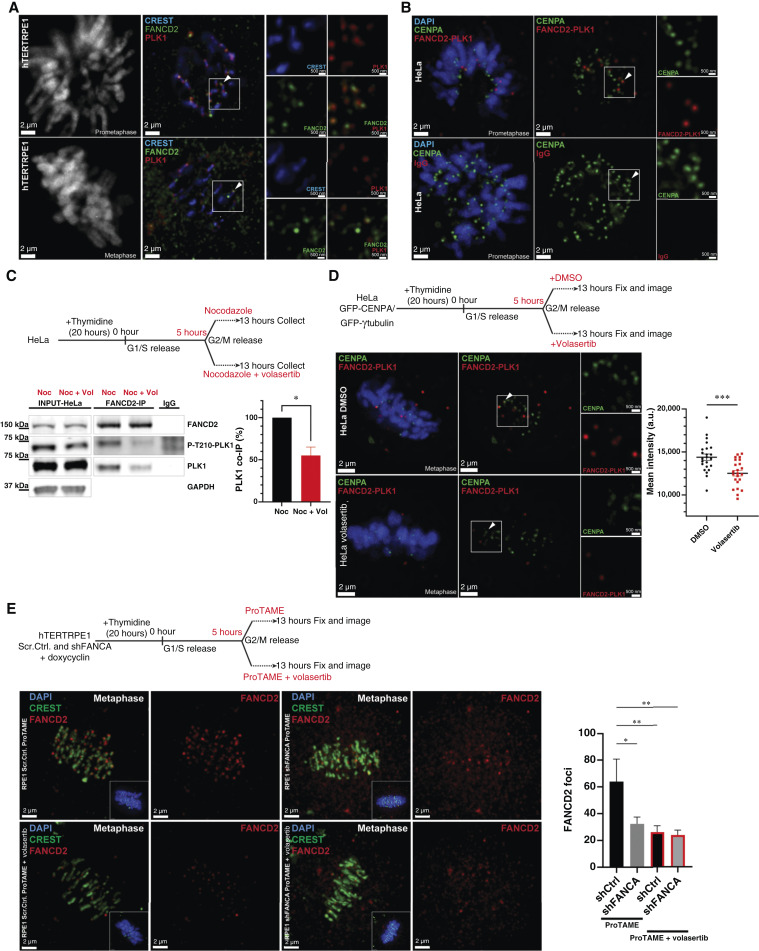
FANCD2 localizes to mitotic centromeres in a PLK1-dependent manner. **A,** Representative IF images show FANCD2 (green) and PLK1 (red) colocalizing at centromeres (CREST, blue) during prometaphase and metaphase in hTERT-RPE1 cells. Scale bars on the left image panels are 2 μm, and scale bars on the right top and bottom are 500 nm. All experiments were repeated a minimum of 3 times. **B,** Representative PLA images shows FANCD2-PLK1 (red) interaction at centromeres (GFP-CENPA, green) in HeLa cells. Scale bars on the left and right image panels are 2 μm and 500 nm, respectively. **C,** Experimental design and a representative Western blot showing IP of endogenous FANCD2 and co-IP of endogenous PLK1 in prometaphase (Noc) HeLa cells. FANCD2-PLK1 interaction is disrupted after PLK1 inhibition (Noc+Vol). Graph represents data of three independent experiments pooled together showing disruption of FANCD2-PLK1 binding after volasertib treatment. Graph shows the mean ± SEM and the *P* value was calculated by an unpaired *t* test. **D,** Experimental design and representative PLA images showing reduced FANCD2–PLK1 (red) interaction at centromeres (GFP-CENPA, green) in HeLa cells. Scale bars on the left and right image panels are 2 μm and 500 nm, respectively. **E,** Experimental design and representative IF images showing loss of localization of FANCD2 (green) at centromeres (CREST, blue) in shFANCA compared with Scr.Ctrl. mitotic hTERT-RPE1 cells. Volasertib (20 nmol/L) treatment reduced FANCD2 localization irrespective of cellular genotype. Scale bars in the image panels represent 2 μm. Representative graph shows the quantification of FANCD2 foci (*n* ≥ 20 cells per group). Graph shows the mean ± SEM. *P* values were calculated by one‐way ANOVA. CENPA, centromere protein A; DAPI, 4',6-diamidino-2-phenylindole; Noc, nocodazole; Vol, volasertib.

During interphase, FANCD2 functions downstream of FANCA in DNA damage repair (1, 81). As such, we reasoned that FANCD2 function may also be dependent on FANCA during mitosis. To determine whether impaired FANCD2 mitotic function secondary to FANCA loss underlies the hypersensitivity of FANCA-deficient cells to PLK1 inhibition, we began by characterizing its localization during mitosis. Previous studies have shown strong accumulation of FANCD2 on mitotic sister chromatids, where it is involved in promoting mitotic DNA synthesis and plays a role in the response to replication stress (17, 82, 83). Using IF microscopy in unperturbed HeLa (Supplementary Fig. S6A and S6B) and hTERT-RPE1 cells, we observed FANCD2 localization to key mitotic structures, including the centrosomes and centromeres. Strikingly, we found PLK1 colocalized with FANCD2 on these subcellular structures (Fig. 6A). Utilizing PLA-IF, we determined that FANCD2 and PLK1 localize within 40 nm of each other on mitotic centromeres in HeLa cells (Fig. 6B). Importantly, we also observed colocalization of PLK1 with both FANCA and FANCD2 at prometaphase centromeres in a patient-derived lymphoblastoid cell line, validating these mitotic interactions in human hematopoietic cells (Supplementary Fig. S7A and S7B). To validate interaction between FANCD2 and PLK1, we evaluated co-IP of PLK1 with endogenous FANCD2 from HeLa cells. PLK1 co-immunoprecipitated with endogenous FANCD2 in mitotic HeLa cells. Interestingly, PLK1 inhibition with volasertib (20 nmol/L) reduced FANCD2-PLK1 co-IP by at least two-fold (Fig. 6C). Accordingly, decreased FANCD2-PLK1 interaction was observed by PLA-IF following volasertib treatment (Fig. 6D). In line with these results, we also observed loss of FANCD2 at centromeres in FANCA*-*knockdown hTERT-RPE1 cells compared with control, suggesting that FANCD2 localization to the centromere is, in part, dependent on FANCA. Strikingly, volasertib treatment also significantly reduced the FANCD2 levels at centromeres independently of FANCA status (Fig. 6E). We did not observe a change in active PLK1 levels, as indicated by T210 phosphorylation, in shFANCA cells compared with control cells (Supplementary Fig. S8A), suggesting that FANCD2 localization to the centromeres may be dependent on both FANCA and PLK1 kinase activity. Interestingly, we did not observe a decrease in FANCD2 localization to MMC-induced γH2AX foci in interphase cells or change in FANCD2 monoubiquitination status after PLK1 inhibition (Supplementary Fig. S8B and S8C). Together, this set of data demonstrates a key role for PLK1 in the recruitment of FANCD2 to mitotic centromeres but not to MMC-induced γH2AX foci.

## Discussion

Although prognosis for patients with AML has dramatically improved over the last several decades, the overall 5-year survival for adults with AML remains approximately 30% (Surveillance Research Program, NCI SEER*Stat software version 12). Importantly, this improved survival can be attributed, in large part, to an increased understanding of the clinical, molecular, and cytogenetic heterogeneity of AML, leading to the development of AML subset–specific therapeutic strategies. Further improvement in patient outcomes will be dependent on the continued characterization of AML subsets conferring increased sensitivity to specific targeted therapies (84, 85). Our work identifies novel synthetic lethal interactions between PLK1 and loss of FA pathway proteins in AML. Whereas this study focuses on PLK1 inhibition to target AMLs with FA pathway disruptions, somatic disruption of the FA pathway is frequently observed in other sporadic malignancies. According to The Cancer Genome Atlas database, approximately 55% of bladder cancers and 35% of head and neck squamous cell carcinomas harbor somatic mutation of FA genes (1). Thus, our findings warrant future preclinical evaluation of PLK1 inhibition as a broad synthetic lethal strategy for treating AML and other malignancies with FA pathway mutations.

PLK1 inhibitors including volasertib and onvansertib have shown some promise in clinical trials evaluating their efficacy for the treatment of AML (38, 40, 86). However, their achieving optimal therapeutic success has been hindered by toxicity and the inability to stratify patients most likely to benefit from PLK1-targeting agents. Thus, the identification of mutations that confer sensitivity to PLK1 inhibition is critical for identifying patients most likely to respond to these drugs at reduced dosages. Several studies have sought to identify genetic alterations that sensitize tumors to PLK1 inhibition. Collectively, these studies have linked disruption of several genes to hypersensitivity to PLK1 inhibition, including *TP53*, *KRAS*, *NF1*, and *CCNE1* (71, 72, 87, 88). As part of the clinical trial evaluating onvansertib as a combination therapy agent for AML, mutations within splicing factors *SRSF2* and *SF3B1* were found to correlate positively with patient response (41). Through retrospective analysis of the RNA-seq data acquired from this trial, we noted that, strikingly, the best response was observed in a patient with three mutations impacting the FA pathway. Although the small sample size and relative rarity of FA pathway mutations do not allow us to draw statistical conclusions regarding the utility of these mutations as molecular predictors of response, this finding does lend preliminary clinical support to our hypothesis. Validation of this synthetic lethal interaction was confirmed through functional *in vitro* studies showing abrogation of FANCA function conferred hypersensitivity to PLK1 inhibition in AML cell lines. Likewise, our *in vitro* assessment of volasertib sensitivity across a panel of primary AML samples from patients cared for at our institution demonstrated that increased mutational burden of the FA pathway is associated with greater sensitivity to PLK1 inhibition. Together, these findings suggest that damaging mutations in the FA pathway and associated genes may predict tumor response to PLK1-targeting agents. Furthermore, our findings in primary murine HSPCs demonstrate that PLK1 inhibition significantly impairs the colony-forming ability of FANCA-deficient progenitors but not their WT counterparts, validating the FA-PLK1 synthetic lethal interaction in a primary congenic background and corroborating work by others who have demonstrated resistance of normal human hematopoietic progenitors to PLK1 inhibition relative to leukemic cells (39). Whereas this selective targeting of FA-deficient HSPCs by PLK1 inhibition may be beneficial in the setting of sporadic AML, it suggests that PLK1 inhibitors, much like DNA-damaging agents (1), may induce high toxicities in patients with germline FA mutations. Further preclinical studies are warranted to assess the *in vivo* therapeutic efficacy of PLK1 inhibition against FA-deficient tumors in the setting of germline FA mutations.

**Figure 7:**
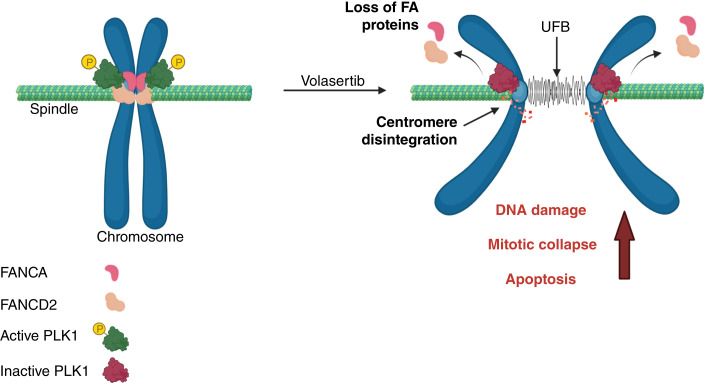
Proposed mechanistic model of PLK1 hypersensitivity in FA-deficient cells. Our findings support a model wherein active PLK1 facilitates localization of FANCD2 to mitotic centromeres to protect centromere integrity (left). In contrast, PLK1 inhibition leads to loss of FANCD2 protein on the centromeres, exacerbating compromised centromere integrity and impaired mitotic fidelity in the setting of FA pathway deficiency (right). The resulting mitotic collapse causes genomic instability, accumulation of DNA damage, and ultimately cell death through apoptosis.

Interrogation of the mechanisms underpinning the synthetic lethal interaction between FANCA and PLK1 revealed increased metaphase collapse, polyploidy, and apoptosis in FANCA-deficient cells after low-concentration volasertib treatment. Relative to untreated cells, we observed an increase in ultrafine bridges and mitotic DNA damage in volasertib-treated cells independent of FANCA status, which may underlie the increased subsequent mitotic failure in FANCA-deficient cells, which are likely unable to repair this damage. Furthermore, we demonstrated FANCD2–PLK1 interaction at mitotic centromeres and found that PLK1 inhibition abrogated localization of FANCD2 to mitotic centromeres. These findings support a model in which interaction with PLK1 facilitates localization of FANCD2 to mitotic centromeres, thereby preserving centromere integrity and subsequent mitotic fidelity (Fig. 7). This model is consistent with studies that have independently demonstrated roles for PLK1 (80, 89) and FA proteins in the maintenance of centromere integrity (90–92). Loss of these FA proteins therefore creates unique vulnerabilities in mitosis that render them highly susceptible to PLK1 inhibition. Whereas our work centers around the mitotic mechanisms underlying synthetic lethality between PLK1 and FA pathway members, increasing evidence implicates PLK1 in homologous recombination–mediated DNA repair (93–95). Interestingly, PLK1 has recently been shown to facilitate optimal activation of the recombinase RAD51 (also known as FANCR) at DNA double-stranded breaks (93). Thus, it is possible that the interplay between PLK1 and the FA pathway in DDR may additionally contribute to this synthetic lethality. This notion is supported by recent work demonstrating that centromeres are hotspots for mitosis-induced breakage following replication stress. Notably, this work identified multiple FA pathway proteins, including FANCD2, in a screen for proteins that co-immunoprecipitated with centromeric chromatin and maintain centromere integrity (96), providing independent validation of our observation of FANCD2 at mitotic centromeres and supporting our model of PLK1–FANCD2-mediated centromere preservation. FA proteins, including FANCD2 and FANCI, have been implicated in the resolution of R-loops (90, 97–99), DNA–RNA hybrid structures that cause replication stress, give rise to UFBs, and cause centromere instability (17, 100–102). Taken together with our findings, these data suggest that disruption of PLK1-mediated FANCD2 recruitment to sites of centromere damage during mitosis compromises chromosome segregation, resulting in mitotic failure.

Whereas this work provides mechanistic insight into poorly understood mitotic functions of the FA pathway and implicates PLK1 inhibition as an attractive strategy for the treatment of FA-disrupted AMLs, we note key limitations to this study. First, the baseline sensitivity and heterogeneous mutational landscape of the AML cell lines and primary samples utilized make it difficult to elucidate the contribution of individual genetic alterations to drug response. However, this limitation reflects real-world clinical challenges presented by the genetic variability among individual tumors and subclones of a single tumor, which confound AML treatment and underlie its poor prognosis, underscoring the need for continued preclinical studies that integrate genetic data with functional evaluation (Res Sq 2023.rs.3.rs-3516536/v1; refs. 103, 104). Likewise, *in vivo* studies are needed to evaluate the therapeutic efficacy of drug sensitizations demonstrated *in vitro*, as well as the assessment of synergy with standard chemotherapeutic agents. A second study limitation is the small sample size of primary AMLs tested for volasertib sensitivity, which precludes robust statistical evaluation of FA mutations as predictors of drug response. Additionally, our study does not directly assess the effect of heterozygous FA mutations on sensitivity to PLK1 inhibition. Although shRNA-mediated gene knockdown does not completely deplete protein product of the targeted gene, this tool may not recapitulate the phenotype of heterozygous FA loss. Likewise, whether the mutation of multiple FA genes has an additive impact on PLK1 dependency should be directly assessed through the generation of isogenic cell lines.

This cumulative body of evidence demonstrates a functional relationship between PLK1 and the FA pathway at mitotic centromeres, highlighting the underappreciated contributions of this classic DNA repair pathway in the process of cell division. Further detailed dissection of the mitotic functions of the FA pathway will be key to understanding the molecular pathogenesis of FA as well as the identification of therapeutically targetable vulnerabilities in FA-disrupted malignancies. It was through careful dissection of FA-directed DNA repair mechanisms that PARP inhibition was identified as a therapeutic strategy for BRCA-deficient cancers (105), representing a historic breakthrough in precision cancer treatment. Likewise, exploration of the understudied roles of FA proteins beyond DNA damage repair may reveal novel synthetic lethal strategies for the treatment of FA-disrupted cancers. Collectively, these findings provide a preclinical rationale for future biomarker-driven clinical trials evaluating PLK1 inhibitors as a targeted therapeutic strategy for AMLs with genetic disruption of the FA pathway.

## Supplementary Material

Supplementary Data STAR table

Supplementary Data Supp Dataset 1

Supplementary Data Supp Dataset 2

Supplementary Data Supp Dataset 3

Supplementary Data Supp Dataset 4

Supplementary Data Supp Dataset 5

Supplementary Data Supp Dataset 6

Supp fig 1 FA pathway-mutated cancer cell lines have increased dependency on PLK1.

Supp fig 2 FANCA-deficiency sensitizes RS4;11 acute lymphoblastic leukemia cell line to PLK1 inhibition.

Supp fig 3 MIB/MS profiling in AML cell lines reveals PLK1 as sole target of Volasertib at low concentrations.

Supp fig 4 FANCA-deficiency sensitizes hematopoietic cells to the PLK1 inhibitor onvansertib.

Supp fig 5 High-confidence non-synonymous FANCA mutations.

Supp fig 6 FANCD2 co-localizes with PLK1 at centromeres in unperturbed mitotic HeLa cells.

Supp fig 7 FANCA and FANCD2 co-localizes with PLK1 at centromeres in unperturbed mitotic hematopoietic cells.

Supp fig 8 The DDR response of FANCD2 is unaffected after PLK1 inhibition.

Supplementary Table 1 Supplemental Table 1

Supplementary Table 2 Supplemental Table 2
