# Supplementary figures and images for: PLK1 Inhibition Induces Synthetic Lethality in Fanconi Anemia Pathway–Deficient Acute Myeloid Leukemia
Source: Cancer Res Commun. 2025 Apr 21;5(4):648–67. doi: 10.1158/2767-9764.CRC-24-0260 (PMC12011380; doi:10.1158/2767-9764.CRC-24-0260)

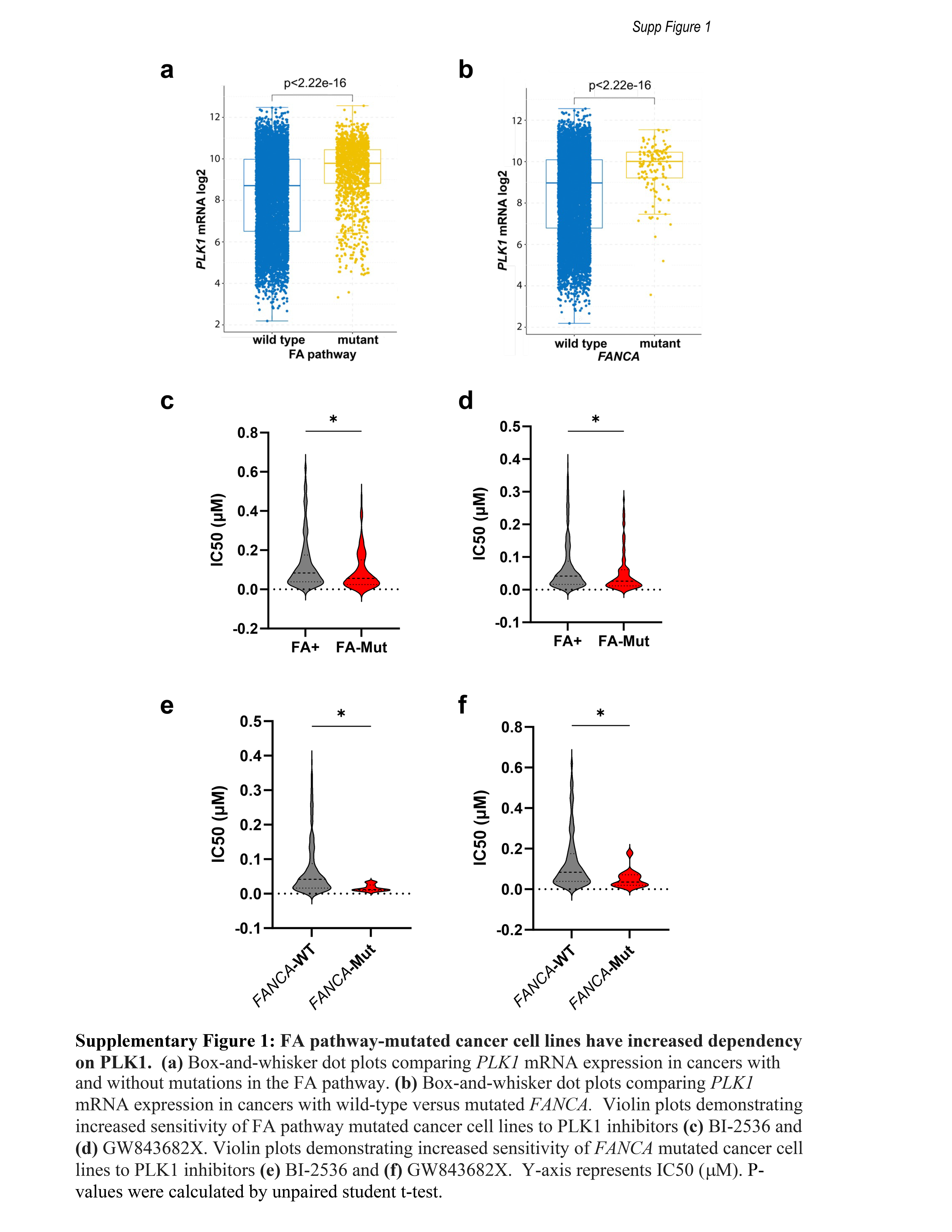

Supplement: Supp fig 1 — FA pathway-mutated cancer cell lines have increased dependency on PLK1. [file crc-24-0260_supp_fig_1_suppsf1.png]

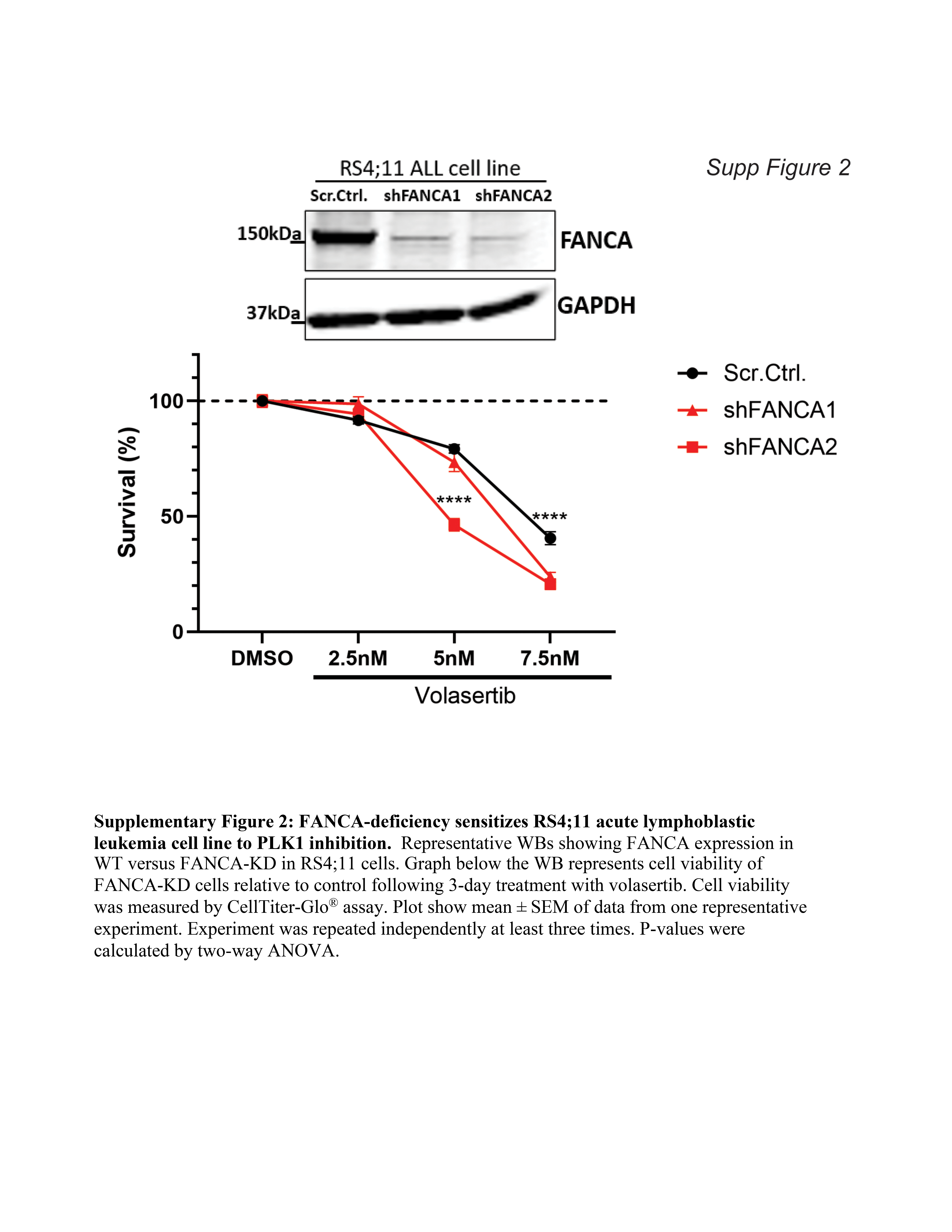

Supplement: Supp fig 2 — FANCA-deficiency sensitizes RS4;11 acute lymphoblastic leukemia cell line to PLK1 inhibition. [file crc-24-0260_supp_fig_2_suppsf2.png]

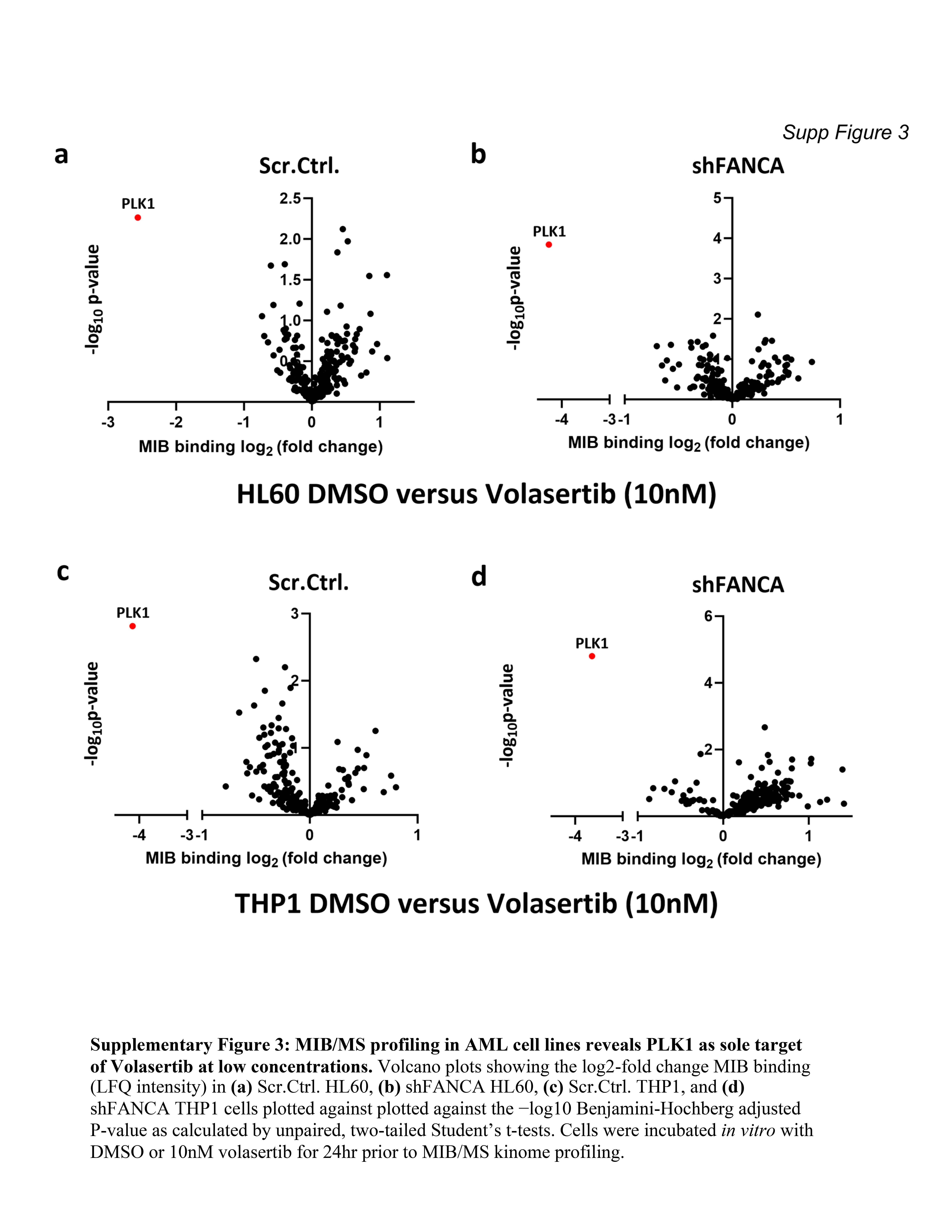

Supplement: Supp fig 3 — MIB/MS profiling in AML cell lines reveals PLK1 as sole target of Volasertib at low concentrations. [file crc-24-0260_supp_fig_3_suppsf3.png]

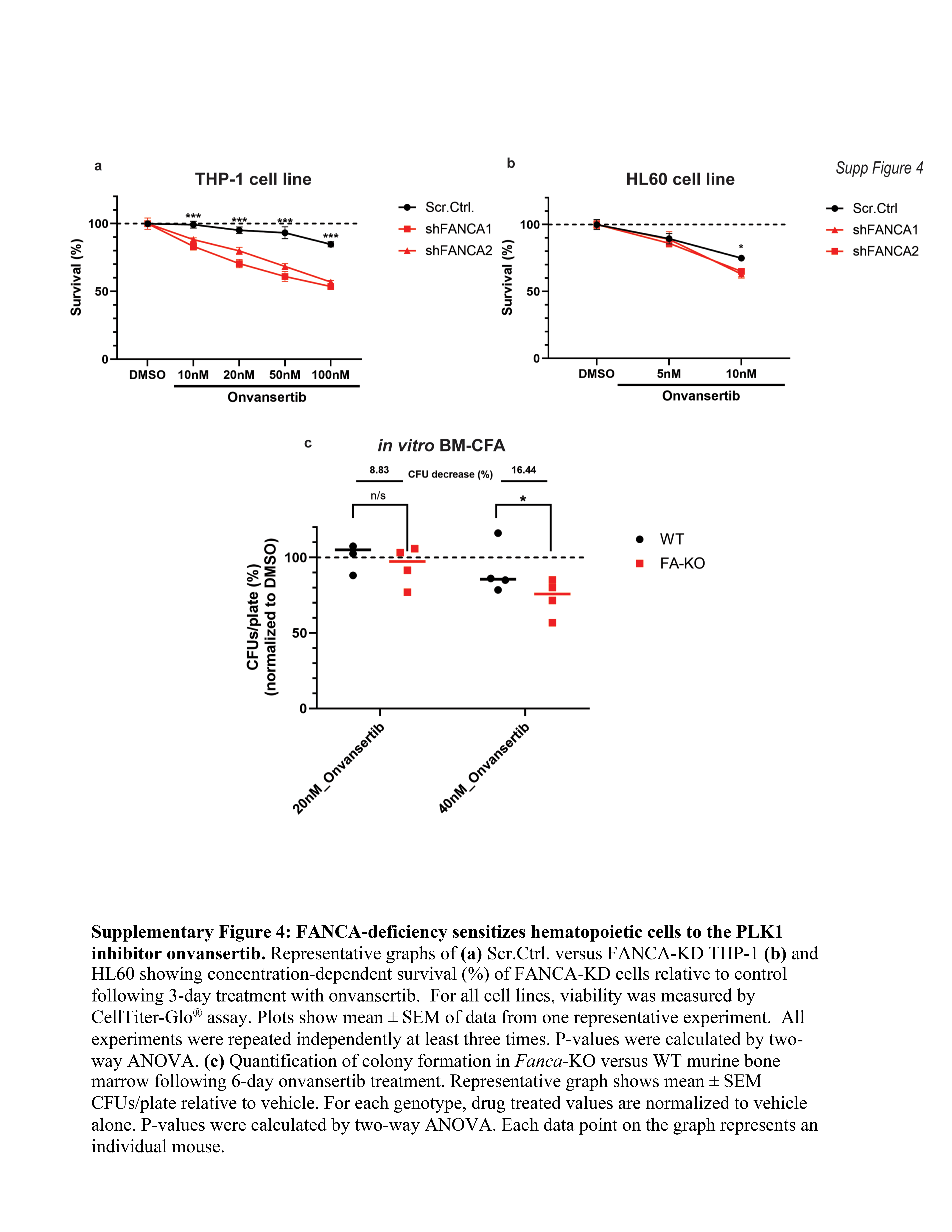

Supplement: Supp fig 4 — FANCA-deficiency sensitizes hematopoietic cells to the PLK1 inhibitor onvansertib. [file crc-24-0260_supp_fig_4_suppsf4.png]

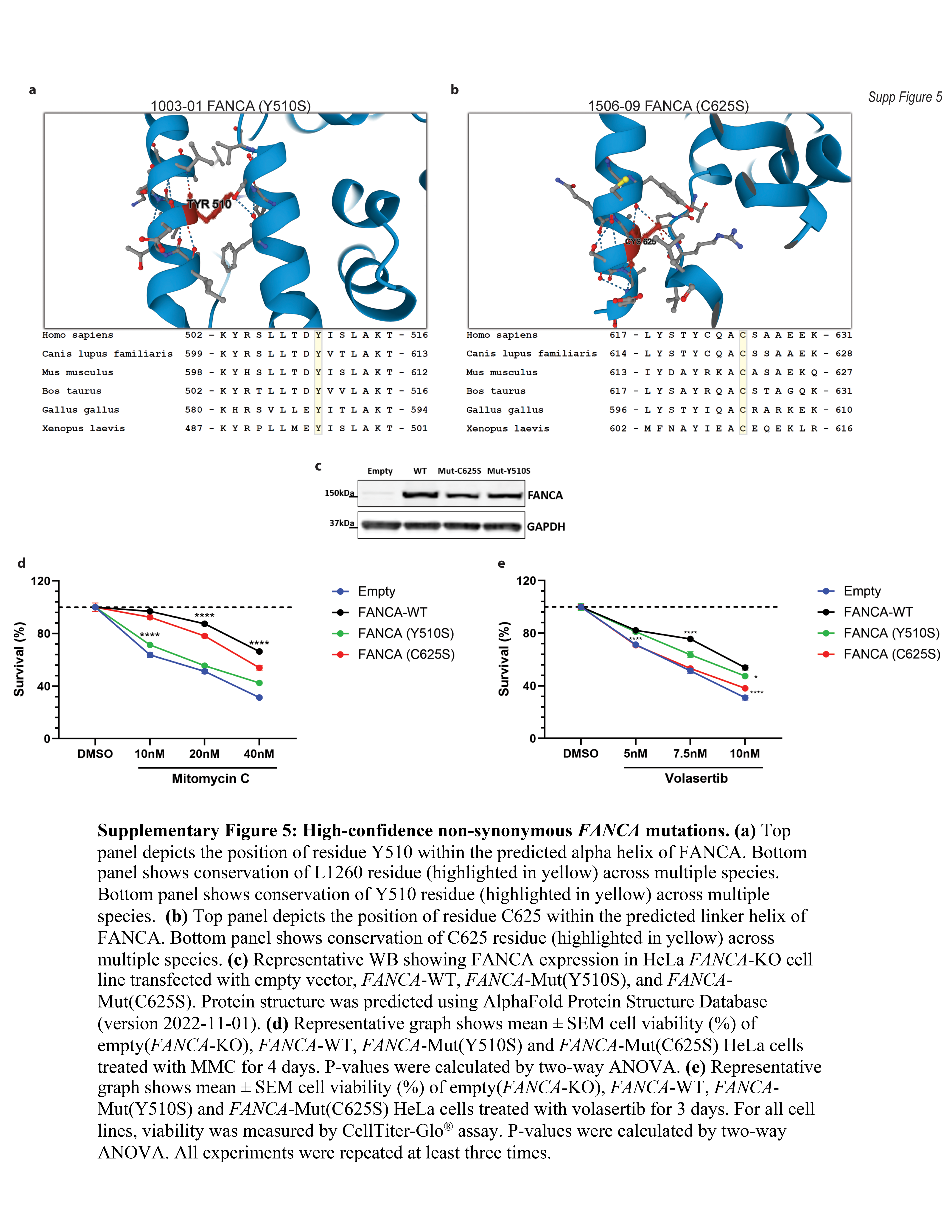

Supplement: Supp fig 5 — High-confidence non-synonymous FANCA mutations. [file crc-24-0260_supp_fig_5_suppsf5.png]

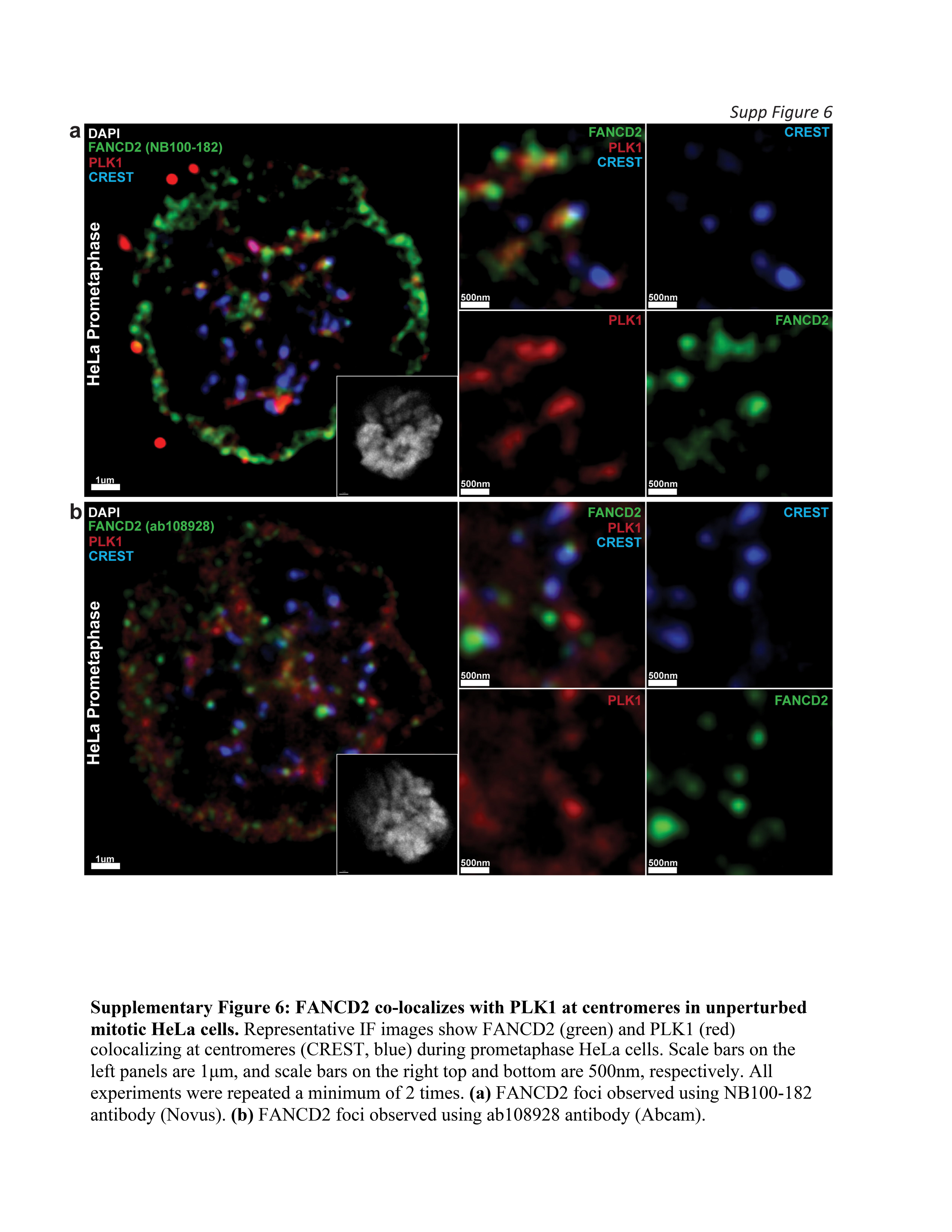

Supplement: Supp fig 6 — FANCD2 co-localizes with PLK1 at centromeres in unperturbed mitotic HeLa cells. [file crc-24-0260_supp_fig_6_suppsf6.png]

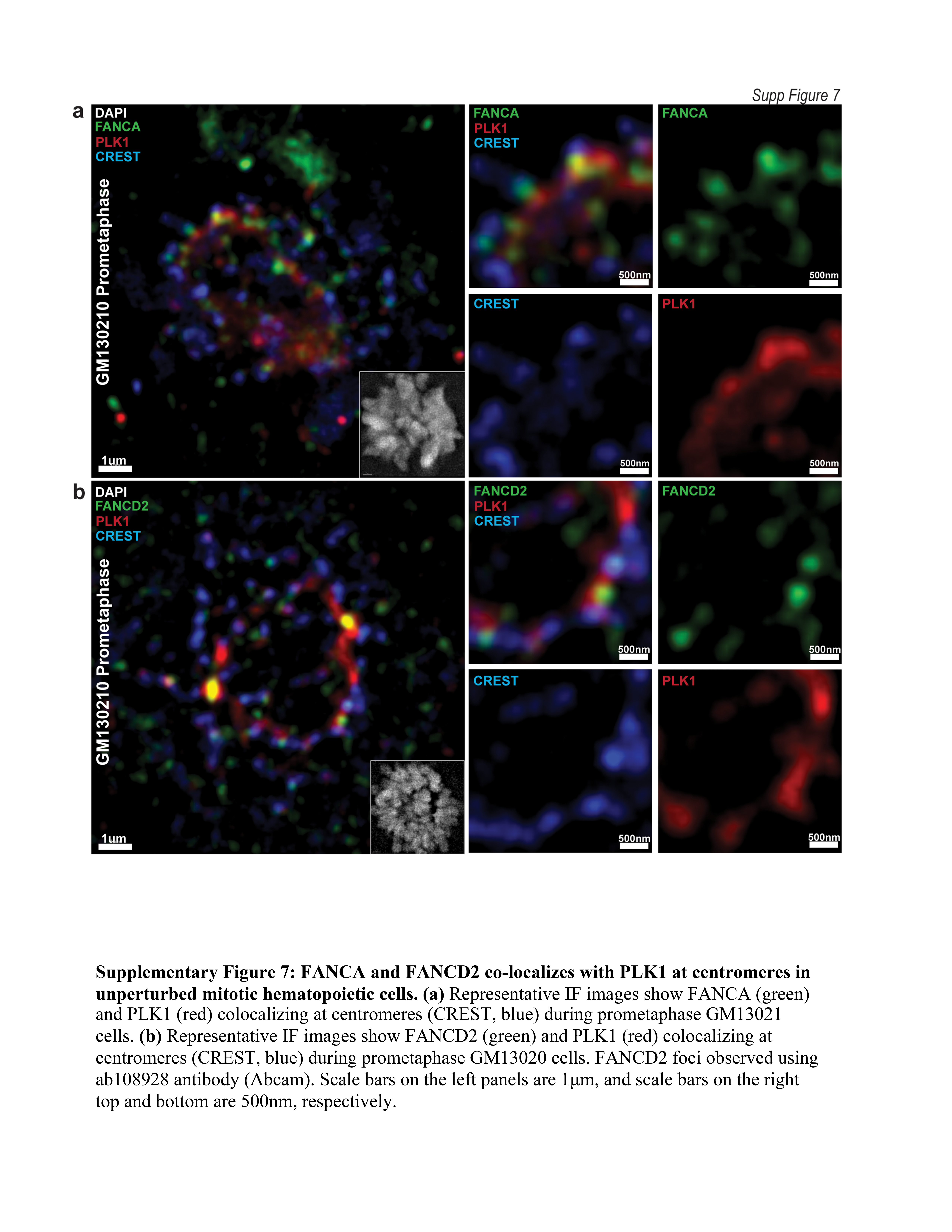

Supplement: Supp fig 7 — FANCA and FANCD2 co-localizes with PLK1 at centromeres in unperturbed mitotic hematopoietic cells. [file crc-24-0260_supp_fig_7_suppsf7.png]

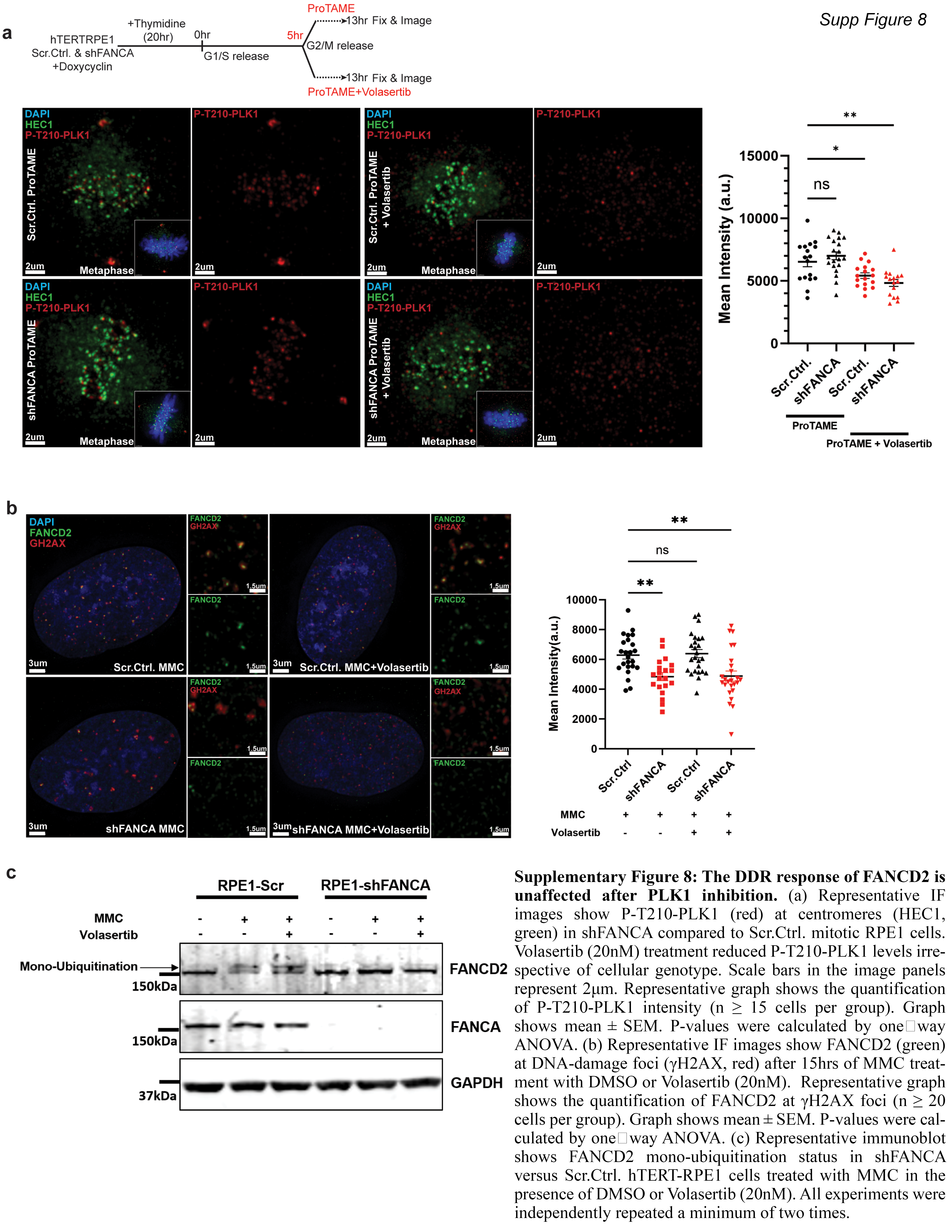

Supplement: Supp fig 8 — The DDR response of FANCD2 is unaffected after PLK1 inhibition. [file crc-24-0260_supp_fig_8_suppsf8.png]
