# Supplementary material for: PLK1 Inhibition Induces Synthetic Lethality in Fanconi Anemia Pathway–Deficient Acute Myeloid Leukemia
Source: Cancer Res Commun. 2025 Apr 21;5(4):648–67. doi: 10.1158/2767-9764.CRC-24-0260 (PMC12011380; doi:10.1158/2767-9764.CRC-24-0260)
Supplement: Supplementary Table 1 — Supplemental Table 1 [file crc-24-0260_supplementary_table_1_suppst1.pdf]

Gene	Company	The RNAi Consortium (TRCN)	Sequence
**FANCA**	MISSION® Lentiviral shRNA	TRCN0000118983	GCACAGGAAATGAGGATATTA
		TRCN0000291182	CAGAGTTCTTTGTTGCTTGAA
**FANCG**		TRCN0000082859	GCCAAAGTCTTGTGACCTGTA
		TRCN0000082860	GCCTCTAAGGATCTGCTGTTA
**FANCD2**		TRCN0000082838	CGTCTATTAGATTGGAGGATT
		TRCN0000082839	GCGTCCATTACTTGCAGAATT
**FANCE**		TRCN0000118817	CCTGAGAAGTTCAGTGTCTTA
		TRCN0000118821	GATATGCCAGAGGAACCTGAT
**FANCS**		TRCN0000010305	AGAATCCTAGAGATACTGAA
		TRCN0000009823	TATAAGACCTCTGGCATGAAT

Supp Table 1: Sequences of shRNA oligonucleotides used for gene knockdown.
