# Supplementary material for: PLK1 Inhibition Induces Synthetic Lethality in Fanconi Anemia Pathway–Deficient Acute Myeloid Leukemia
Source: Cancer Res Commun. 2025 Apr 21;5(4):648–67. doi: 10.1158/2767-9764.CRC-24-0260 (PMC12011380; doi:10.1158/2767-9764.CRC-24-0260)
Supplement: Supplementary Table 2 — Supplemental Table 2 [file crc-24-0260_supplementary_table_2_suppst2.pdf]

Antibody Target	Catalog Number	Application / Molecular Weight
**BLM**	SCBT Cat# sc-7790, RRID: AB_2243489	IF
**BRCA1**	CST Cat# 9010, RRID: AB_2228244	WB (MW: 220kDa)
**CREST**	Calbiotech Cat# HCT-0100, RRID: AB_2744669	IF
**Cyclin B1**	CST Cat# D5C10, RRID: AB_3075525	WB (MW: 55kDa)
**FAN1**	Abcam Cat# ab68572, RRID: AB_1310472	WB (MW: 114kDa)
**FANCA**	Abcam Cat# ab97578, RRID: AB_10680297	IP (MW: 160kDa)
**FANCA**	Abcam Cat# ab201458, RRID: ab201458	WB, IP, IF (MW: 160kDa)
**FANCD2**	Abcam Cat# ab108928, RRID: AB_10862535	IP, IF
**FANCD2**	Novus Cat# NB100-182, RRID: AB_10002867	IF
**FANCD2**	SCBT Cat# sc-20022, RRID: AB_2278211	WB (MW: 160kDa)
**FANCE**	SCBT Cat# sc-398558, RRID: sc-398558	WB (MW: 59kDa)
**FANCG**	Abcam Cat# ab151516, RRID: ab151516	WB (MW: 69kDa)
**GAPDH**	CST Cat# 14C10, RRID: AB_1903993	WB (MW: 36kDa)
**γH2AX**	Sigma Cat# JBW301, RRID: AB_2924829	IF
**PLK1**	Abcam Cat# ab17056, RRID: AB_443612	WB (MW: 68kDa)
**pT210 PLK1**	Abcam Cat# ab278768, RRID: ab278768	WB (MW: 68kDa)
**pT210 PLK1**	CST Cat# 5472T, RRID: CST; 5472T	IF
**RPA70**	Abcam Cat# ab79398, RRID: AB_1603759	IF
**TOP2a**	Origene Cat# UM800042, RRID: AB_2629154	IF
**β-actin**	Sigma Cat# A5441, RRID: AB_476744	WB (MW: 42kDa)

Supp Table 2: Primary antibodies used in Western Blots (WB), Immunofluorescence (IF) and Immunoprecipitation (IP).
